# Anticancer Activity of 2,3′-Dihydroxy-5′-Methoxystilbene Against NSCLC Cell Lines Through AKT-Dependent Mechanisms: A Comprehensive In Vitro and Computational Analysis

**DOI:** 10.3390/ijms27020719

**Published:** 2026-01-10

**Authors:** Phisit Pouyfung, Nonthalert Lertnitikul, Noriyoshi Ogino, Achitphol Chookaew, Varisa Pongrakhananon, Piriya Chonsut, Natthaporn Sueangoen, Suwichak Chaisit

**Affiliations:** 1 Department of Occupational Health and Safety, School of Public Health, Walailak University, Nakhon Si Thammarat 80160, Thailand; phisit.po@mail.wu.ac.th; 2 Biomass and Oil Palm Center of Excellence, Walailak University, Nakhon Si Thammarat 80160, Thailand; 3 Department of Pharmacognosy and Pharmaceutical Botany, Faculty of Pharmaceutical Sciences, Chulalongkorn University, Bangkok 10330, Thailand; nonthalert.l@chula.ac.th; 4 Animal Models of Chronic Inflammation Associated Diseases for Drug Discovery Research Unit, Chulalongkorn University, Bangkok 10330, Thailand; 5 Third Department of Internal Medicine, School of Medicine, University of Occupational and Environmental Health, Iseigaoka 1-1, Yahatanishi-ku, Kitakyushu 807-8555, Japan; n-ogino@med.uoeh-u.ac.jp; 6 Division of Hematopoiesis, Joint Research Center for Human Retrovirus Infection, Graduate School of Medical Sciences, Kumamoto University, Honjo, Chuou-ku, Kumamoto 860-0811, Japan; achitphol.ch@gmail.com; 7 Department of Pharmacology and Physiology, Faculty of Pharmaceutical Sciences, Chulalongkorn University, Bangkok 10330, Thailand; varisa.p@pharm.chula.ac.th; 8 Center of Excellence in Preclinical Toxicity and Efficacy Assessment of Medicines and Chemicals, Chulalongkorn University, Bangkok 10330, Thailand; 9 Department of Applied Thai Traditional Medicine, School of Medicine, Walailak University, Nakhon Si Tammarat 80160, Thailand; piriya.ch@wu.ac.th; 10 Research Laboratory Section, Offices of Health Science Research, Faculty of Medicine Ramathibodi Hospital, Mahidol University, Bangkok 10400, Thailand; natthaporn.sue@mahidol.ac.th; 11 Department of Chemical Engineering and Pharmaceutical Chemistry, School of Engineering and Technology, Walailak University, Nakhon Si Thammarat 80160, Thailand

**Keywords:** 2,3′-dihydroxy-5′-methoxystilbene, non-small cell lung cancer (NSCLC), AKT/GSK3β signaling pathway, cell apoptosis, chemosensitization

## Abstract

Lung cancer remains a major clinical challenge, with therapy resistance in non-small-cell lung cancer (NSCLC) driving the search for novel selective agents. This study demonstrates that 2,3′-dihydroxy-5′-methoxystilbene exhibits significant anticancer activity in NSCLC cell lines (A549, H23, and H460) while displaying substantially lower toxicity toward normal NIH/3T3 fibroblasts. The compound reduced the viability of H23 and H460 cells after 48 h. (IC50: 23.39 ± 3.27 μM and 24.20 ± 2.61 μM, respectively), with NIH/3T3 cells remaining comparatively resistant (IC50 > 100 μM). At 25 μM, it suppressed proliferation by approximately 40% in H23, 30% in H460, and 20% in A549 cells, and dose-dependently impaired colony formation and migration, leading to near-complete migration arrest in H460 cells. Apoptosis induction peaked at 19% in H23, 17% in H460, and 8% in A549 cells at 25 μM. Mechanistic studies and molecular modeling revealed AKT-dependent activity, with decreased p-AKT and p-GSK3β levels (0.70 and 0.75 in H23; 0.65 and 0.70 in H460 at 25 μM), without changes in total protein expression. Combination treatment with cisplatin yielded synergistic effects in A549 (CI = 0.83) and H460 (CI = 0.94) cells, but antagonistic effects in H23 cells (CI = 1.32). These findings identify 2,3′-dihydroxy-5′-methoxystilbene as a selective AKT-targeting stilbene with promising anticancer potential and context-dependent chemosensitizing activity in NSCLC cells.

## 1. Introduction

Lung cancer remains a critical global health burden and is the leading cause of cancer-related mortality worldwide, accounting for approximately 1.8 million deaths annually, representing nearly one-fifth of all cancer fatalities [1,2]. While tobacco smoking constitutes the predominant risk factor, other significant etiological determinants include occupational and environmental carcinogens (e.g., radon and asbestos), genetic susceptibility, and chronic pulmonary diseases [3]. Lung cancer is histologically categorized into non-small-cell lung cancer (NSCLC) and small-cell lung cancer (SCLC), which are distinguished by their divergent pathological features and therapeutic requirements. NSCLC accounts for approximately 85% of all lung cancer cases and comprises three principal subtypes: adenocarcinoma, squamous cell carcinoma, and large-cell carcinoma [4]. Despite substantial advancements in targeted therapies and immunotherapies, the clinical outcomes of NSCLC remain compromised by significant therapeutic challenges. Acquired drug resistance poses a critical challenge, particularly in the case of Epidermal Growth Factor Receptor (EGFR) tyrosine kinase inhibitors (TKIs). Notably, the EGFR T790M secondary mutation is the predominant mechanism of resistance, accounting for 50–60% of these cases [5,6]. Mechanistically, this mutation involves the substitution of threonine with a bulky methionine residue at position 790 in the EGFR kinase domain. This structural alteration creates steric hindrance that limits TKI binding while enhancing ATP affinity, compromising therapeutic efficacy [7,8]. Furthermore, heterogeneity within the tumor microenvironment (TME) significantly contributes to therapeutic resistance and disease progression. Central to these adaptive mechanisms is the PI3K/AKT signaling pathway, which governs critical cellular processes driving lung tumorigenesis [9,10]. Constitutive hyper-activation of AKT, as evidenced by phosphorylation at Ser473, correlates with poor clinical prognosis and therapeutic resistance in patients with NSCLC [11,12]. Consequently, this signaling cascade facilitates epithelial–mesenchymal transition (EMT), angiogenesis, and chemoresistance. Furthermore, AKT-mediated inactivation of Glycogen Synthase Kinase-3β (GSK3β) via phosphorylation at Ser9 promotes tumor progression by stabilizing oncogenic transcription factors, thereby driving neoplastic proliferation [13,14]. Consequently, pharmacological targeting of the PI3K/AKT/GSK3β signaling axis represents a promising therapeutic strategy for developing novel NSCLC interventions [15,16].

Polyphenolic compounds, including flavonoids and stilbenoids, have attracted considerable scientific interest for their potential in cancer chemoprevention and therapeutics, owing to their intrinsic anti-inflammatory, antioxidant, and antineoplastic activities. These bioactive phytochemicals modulate critical oncogenic signaling networks, including the PI3K/AKT and MAPK/ERK pathways. Recent investigations have elucidated the distinct mechanistic profiles of these subclasses. For instance, eupafolin, a flavonoid isolated from *Salvia plebeia*, inhibits the proliferation, migration, and invasion of NSCLC cells by suppressing the FAK/PI3K/AKT signaling axis and downregulating metastasis-associated biomarkers [17]. Similarly, isalpinine, a flavonoid derived from *Paphiopedilum dianthum*, exhibited anticancer activity against NSCLC cell lines (A549, H23, and H460) by reducing cell viability in a dose- and time-dependent manner. Notably, H23 and H460 cells displayed heightened sensitivity to this compound (IC_50_: 44 μM, 48 h). Furthermore, isalpinine suppressed proliferation, migration, and anchorage-independent growth, while inducing apoptosis mediated by reactive oxygen species (ROS) generation and Bcl-2 downregulation [18]. Stilbenes are a prominent subclass of polyphenolic compounds with substantial therapeutic potential. Both natural and synthetic stilbene derivatives exhibit potent anticancer activity, with their pharmacological efficacy frequently governed by the specific nature and positional arrangement of substituents on the stilbene scaffold [19]. A prototypical example is resveratrol, which has demonstrated extensive bioactivity in various cancer models [19]. Another key analog is pterostilbene, which exerts antitumor effects through epigenetic regulation, including the modulation of DNA methylation and inhibition of histone deacetylases (HDACs). Furthermore, combretastatin A-4, a highly potent stilbenoid, functions by inhibiting tubulin polymerization and disrupting tumor angiogenesis, underscoring the structural versatility of the stilbene scaffold in the development of anticancer therapies [20]. The compound 2,3′-dihydroxy-5′-methoxystilbene, isolated from *Paphiopedilum dianthum* (Orchidaceae), exhibited moderate cytotoxic activity against human breast cancer MCF-7 cells and their doxorubicin-resistant variant (MCF-7/DOX), while maintaining a favorable safety profile in normal NIH/3T3 fibroblasts. Conversely, structurally related stilbene dimers, specifically (E)-2,5′-dihydroxy-2′-(4-hydroxybenzyl)-3′-methoxystilbene and paphiodianthin-A, displayed superior potency against both parental and resistant phenotypes. These findings suggest that specific structural modifications, particularly dimerization, critically influence the structure–activity relationship (SAR) of stilbenoids derived from this species [21]. Although 2,3′-dihydroxy-5′-methoxystilbene exhibits attenuated potency and multidrug-resistance (MDR) reversal efficacy relative to certain dimeric analogs, it maintains distinct selectivity toward MDR breast cancer phenotypes while exhibiting a favorable safety profile in normal cells. Consequently, although current evidence suggests that this compound may not be the most potent derivative within its class, its specific selectivity warrants further pharmacological evaluation. From a mechanistic standpoint, 2,3′-dihydroxy-5′-methoxystilbene is a promising lead compound. Its mono-methoxylated stilbene framework, featuring two phenolic hydroxyl groups, retains the redox-active and hydrogen bonding properties of resveratrol, while slightly enhancing lipophilicity. This combination is advantageous for interacting with kinase ATP-binding sites and influencing PI3K/AKT signaling. Alongside its previously demonstrated selective cytotoxic and chemosensitizing effects on multidrug-resistant breast cancer cells while sparing NIH/3T3 fibroblasts, indicating a favorable therapeutic window, these structural and pharmacological traits strongly support the evaluation of 2,3′-dihydroxy-5′-methoxystilbene, derived from *Paphiopedilum dianthum* as a potential AKT-targeting and redox-modulating agent in NSCLC. Considering the pivotal role of the PI3K/AKT pathway and redox imbalance in NSCLC, these attributes provide a compelling reason to explore 2,3′-dihydroxy-5′-methoxystilbene as a potential AKT-targeting and redox-modulating agent in NSCLC cell lines. Despite these preliminary findings in breast cancer models [21], a significant void remains in the literature regarding the broader antineoplastic spectrum of this compound. However, its pharmacological effects and underlying molecular mechanisms in non-small-cell lung cancer (NSCLC) remain uncharacterized. Given the critical need for novel interventions in NSCLC and the prognostic significance of the PI3K/AKT signaling axis, this study aimed to evaluate the anticancer efficacy of 2,3′-dihydroxy-5′-methoxystilbene. We assessed the ability of these compounds to modulate the key hallmarks of malignancy, including cell viability, long-term proliferation, migration, anchorage-independent growth, and apoptosis induction. Furthermore, this investigation sought to elucidate the molecular mechanisms driving these phenotypic effects, specifically hypothesizing that the observed antitumor activity is mediated through the inhibition of the PI3K/AKT/GSK3β signaling cascade across distinct NSCLC subtypes.

## 2. Results

### 2.1. Effect of 2,3′-Dihydroxy-5′-Methoxystilbene on Cell Toxicity in NSCLC Cell Lines

**Figure 1:**
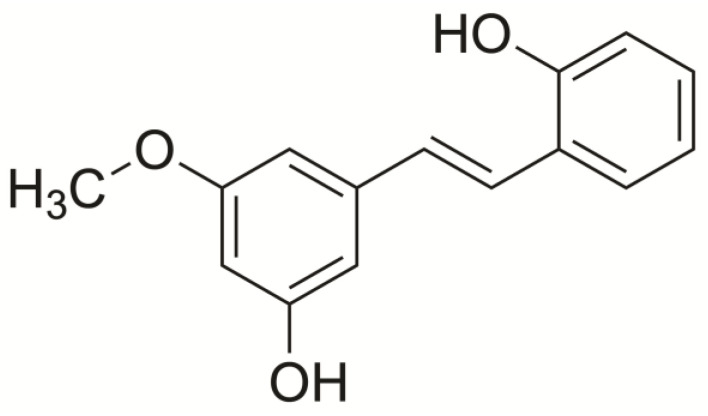
Chemical structure of 2,3′-dihydroxy-5′-methoxystilbene.

**Figure 2:**
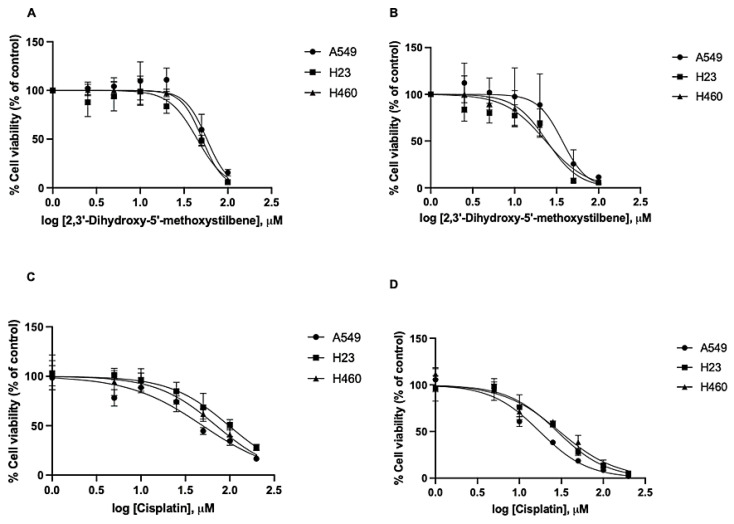
Cytotoxicity of 2,3′-dihydroxy-5′-methoxystilbene and cisplatin in NSCLC cell lines. Cell viability was assessed using the MTT assay. (**A**,**B**) Viability of A549, H23, and H460 cells treated with 2,3′-dihydroxy-5′-methoxystilbene for (**A**) 24 h. and (**B**) 48 h. (**C**,**D**) Comparative cytotoxicity of the positive control, cisplatin, following treatment for (**C**) 24 h and (**D**) 48 h. Data are presented as mean ± SEM (*n* = 3 independent experiments).

To evaluate the antineoplastic potential of 2,3′-dihydroxy-5′-methoxystilbene (Figure 1), we assessed its cytotoxicity against a panel of NSCLC cell lines (A549, H23, and H460) and normal murine fibroblasts (NIH/3T3) using the MTT assay. Dose–response analysis revealed that the compound reduced cell viability in a time- and dose-dependent manner (Figure 2A–D). As detailed in Table 1, at 24 h, 2,3′-dihydroxy-5′-methoxystilbene exhibited moderate cytotoxicity with IC_50_ values of 57.68 ± 4.93 µM (A549), 44.79 ± 5.92 µM (H23), and 51.14 ± 3.91 µM (H460). Notably, at this time point, the stilbene derivative demonstrated superior potency compared to cisplatin in H23 (IC_50_: 97.87 ± 8.99 µM) and H460 (IC_50_: 70.46 ± 6.36 µM) cells. Prolonged exposure (48 h) significantly enhanced the efficacy of 2,3′-dihydroxy-5′-methoxystilbene, reducing the IC_50_ to 23.39 ± 3.27 µM in H23 and 24.20 ± 2.61 µM in H460 cells. Cisplatin generally exhibited higher overall cytotoxicity at 48 h. (IC_50_ range: 12.39–30.12 µM), displaying non-selective toxicity toward normal cells. Crucially, 2,3′-dihydroxy-5′-methoxystilbene maintained a favorable safety profile in NIH/3T3 fibroblasts, with IC_50_ values exceeding 100 µM at both 24 and 48 h. In contrast, cisplatin was highly toxic to normal fibroblasts (IC_50_: 12.39 ± 3.28 µM at 48 h). These results demonstrate that 2,3′-dihydroxy-5′-methoxystilbene exhibits a high degree of selectivity for NSCLC cells, particularly in the H23 and H460 cell lines. Since NIH/3T3 fibroblasts are murine and not lung-derived, this selectivity should be viewed as a preliminary indicator of nontumor toxicity rather than a definitive assessment of safety in normal human lung epithelium.

### 2.2. 2,3′-Dihydroxy-5′-Methoxystilbene Suppresses Proliferation in NSCLC Cells

**Figure 3:**
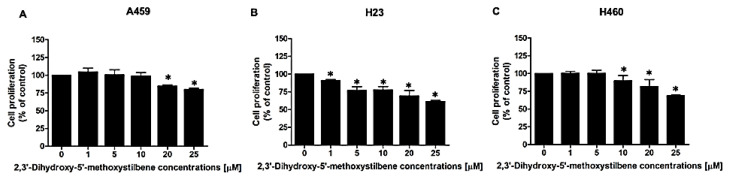
Antiproliferative effects of 2,3′-dihydroxy-5′-methoxystilbene on NSCLC cell lines after 48 h of treatment. Cell proliferation was evaluated using the MTT assay following exposure to increasing concentrations (1, 5, 10, 20, and 25 μM) of 2,3′-dihydroxy-5′-methoxystilbene for 48 h. The analysis was performed on (**A**) A549, (**B**) H23, and (**C**) H460 cell lines. Results are expressed as a percentage of the control group (mean ± SEM; *n* = 3). Statistical significance was assessed using a *t*-test to compare each treatment concentration with the control (* *p* < 0.05).

Sustained proliferation is a hallmark of malignant tumors. To evaluate the antiproliferative efficacy of 2,3′-dihydroxy-5′-methoxystilbene, we assessed the growth rates of NSCLC cell lines (A549, H23, and H460) after incubation with increasing concentrations (1–25 μM) for 48 h. As illustrated in Figure 3, the compound elicited significant dose-dependent suppression of cell growth across all cell lines, although the sensitivity varied according to the cellular context. H23 cells exhibited the highest susceptibility, displaying a marked reduction in proliferation of approximately 40% at 25 µM (Figure 3B). H460 cells exhibited a moderate response, achieving 30% inhibition at the highest concentration (Figure 3C). Conversely, A549 cells were the least responsive (Figure 3A), with significant growth suppression (20%) observed only at 25 µM (*p* < 0.05). These differential response profiles indicate that the antiproliferative efficacy of 2,3′-dihydroxy-5′-methoxystilbene is contingent on cell line-specific molecular determinants, supporting its potential as a targeted therapeutic candidate for lung cancer treatment.

### 2.3. 2,3′-Dihydroxy-5′-Methoxystilbene Attenuates Anchorage-Independent Growth in NSCLC Cells

**Figure 4:**
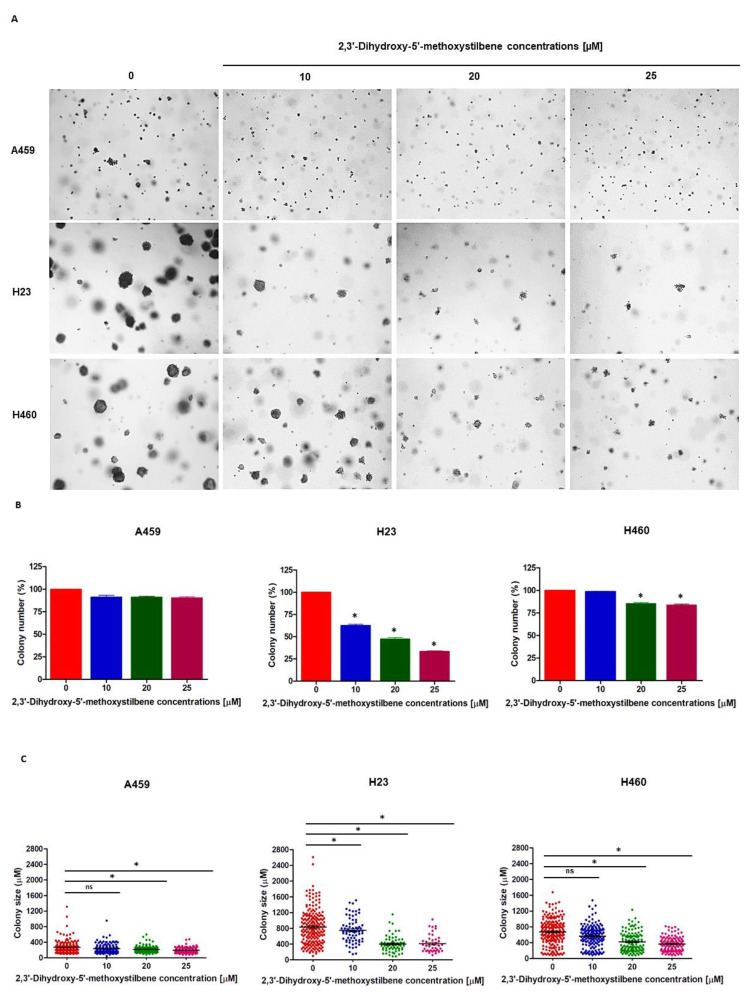
Anti-clonogenic effects of 2,3′-dihydroxy-5′-methoxystilbene on NSCLC cell lines. (**A**) Representative images of colony formation assays showing dose-dependent reduction in colony number and size across A549, H23, and H460 cell lines treated with increasing concentrations (0, 10, 20, 25 μM) of 2,3′-dihydroxy-5′-methoxystilbene for 14 days. (**B**) Quantitative analysis of colony number expressed as percentage of control (mean ± SEM, *n* = 3). (**C**) Colony size distribution analysis showing individual colony measurements across treatment groups. Statistical significance was determined by *t*-test comparing each treatment concentration to vehicle control (* *p* < 0.05, ns = not significant).

Anchorage-independent growth is a hallmark of malignant transformation, indicating resistance to anoikis and metastasis. To evaluate the efficacy of 2,3′-dihydroxy-5′-methoxystilbene in suppressing this phenotype, H23, H460, and A549 cells were cultured in soft agar matrices in the presence or absence of the compound for 14 days. Treatment elicited a significant cell line-dependent impairment of clonogenic survival, as evidenced by reductions in both colony abundance and dimensions (Figure 4A–C). Quantitative analysis revealed that H23 cells were the most sensitive to this compound. A clear dose-dependent inhibition was observed, with colony numbers decreasing to approximately 60%, 45%, and 35% of the control at 10, 20, and 25 μM, respectively (Figure 4B). Concomitantly, statistically significant reductions in colony size were observed beginning at 10 μM, which progressively intensified at higher concentrations (*p* < 0.05; Figure 4C). H460 cells exhibited intermediate sensitivity; significant decreases in colony number were restricted to the 25 μM treatment group (80% of control; *p* < 0.05), whereas significant reductions in colony size were evident at both 20 μM and 25 μM concentrations (Figure 4C). Conversely, A549 cells were largely resistant to anti-clonogenic modulation. Colony counts remained statistically comparable to controls across all concentrations, although a modest but significant reduction in the mean colony size was detected at 25 µM (*p* < 0.05). Collectively, these data demonstrate that 2,3′-dihydroxy-5′-methoxystilbene suppresses anchorage-independent growth, with the magnitude of the effect dependent on the specific cellular context.

### 2.4. Inhibitory Effect of 2,3′-Dihydroxy-5′-Methoxystilbene on Lung Cancer Cell Migration

**Figure 5:**
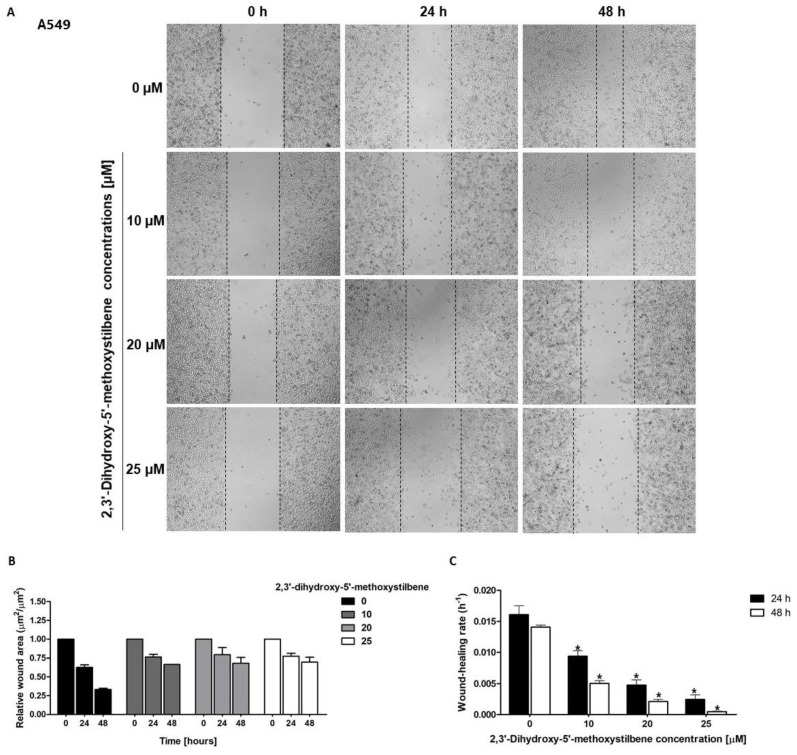

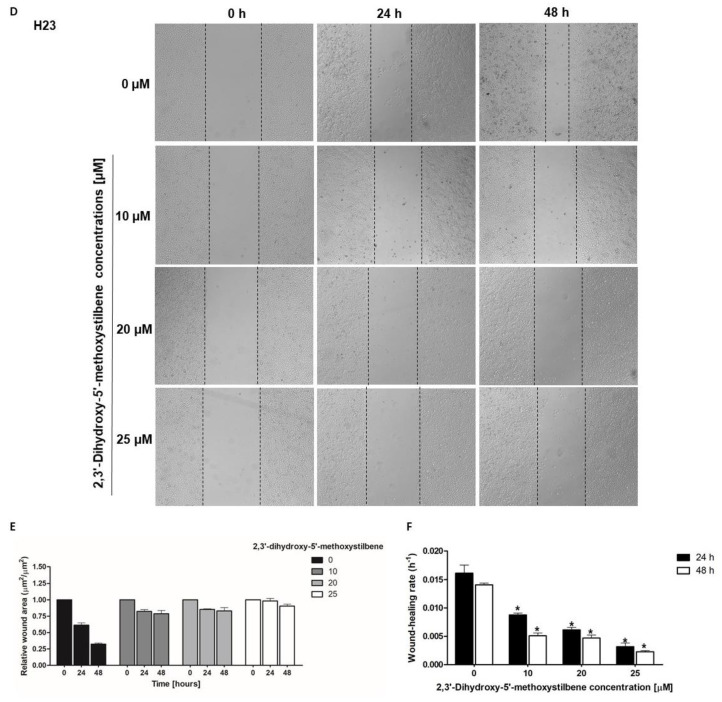

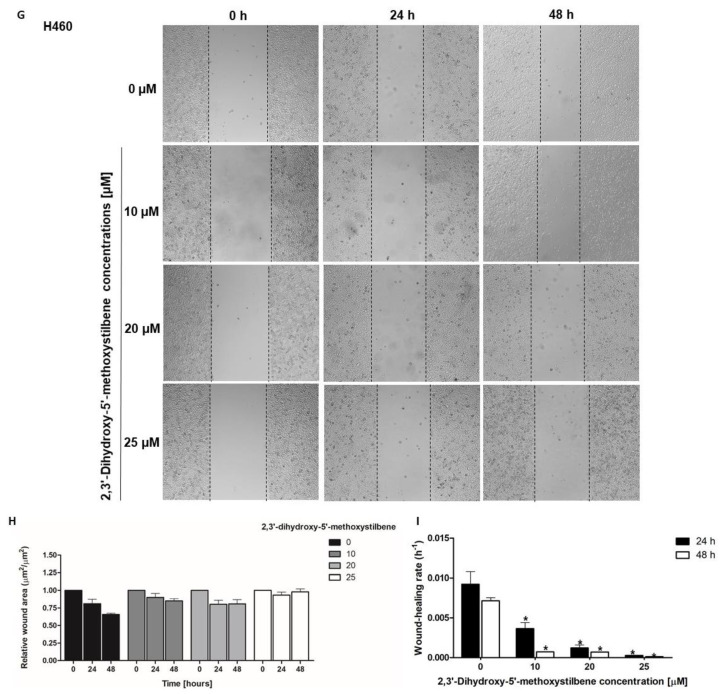
Effect of 2,3′-dihydroxy-5′-methoxystilbene on NSCLC cell migration assessed by wound healing assays. (**A**,**D**,**G**) Representative images of wound closure in A549 (**A**), H23 (**D**), and H460 (**G**) cells treated with increasing concentrations of stilbene (0, 10, 20, 25 μM) for up to 48 h. (**B**,**E**,**H**) Histograms show the relative wound area over time, presented as the mean ± SEM (*n* = 3). (**C**,**F**,**I**) Wound healing rates (h ^− 1^) at 24 and 48 h. for each treatment concentration. Statistical significance was determined by *t*-test vs. control (* *p* < 0.05).

Cell migration is a fundamental prerequisite for metastasis, enabling malignant cells to breach tissue barriers and colonize distant anatomical sites of the body. To evaluate the antimigratory potential of 2,3′-dihydroxy-5′-methoxystilbene, wound healing assays were performed on genetically distinct NSCLC cell lines (A549, H23, and H460) for 48 h (Figure 5). Quantitative analysis revealed a cell line-specific sensitivity to this compound. H460 cells exhibited the most pronounced response to treatment with 25 μM 2,3′-dihydroxy-5′-methoxystilbene, resulting in near-complete migration arrest, with wound closure rates dropping significantly from 0.010 h^−1^ in controls to approximately 0.0001 h^−1^ (Figure 5I; *p* < 0.05). A549 cells displayed intermediate sensitivity, with a concentration-dependent inhibition (Figure 5C). The wound healing rates decreased to 0.005, 0.003, and 0.001 h^−1^ at 10, 20, and 25 μM, respectively, compared to the control rate of 0.017 h^−1^. In contrast, H23 cells were less responsive (Figure 5F), with rates decreasing modestly from 0.016 h^−1^ (control) to 0.003 h^−1^ at the highest concentration. These differential cell migration inhibition results suggest that the anti-metastatic efficacy of 2,3′-dihydroxy-5′-methoxystilbene depends on the specific cellular mutation profile of the target cells.

### 2.5. Effect of 2,3′-Dihydroxy-5′-Methoxystilbene on Oxidative Stress Induction

**Figure 6:**
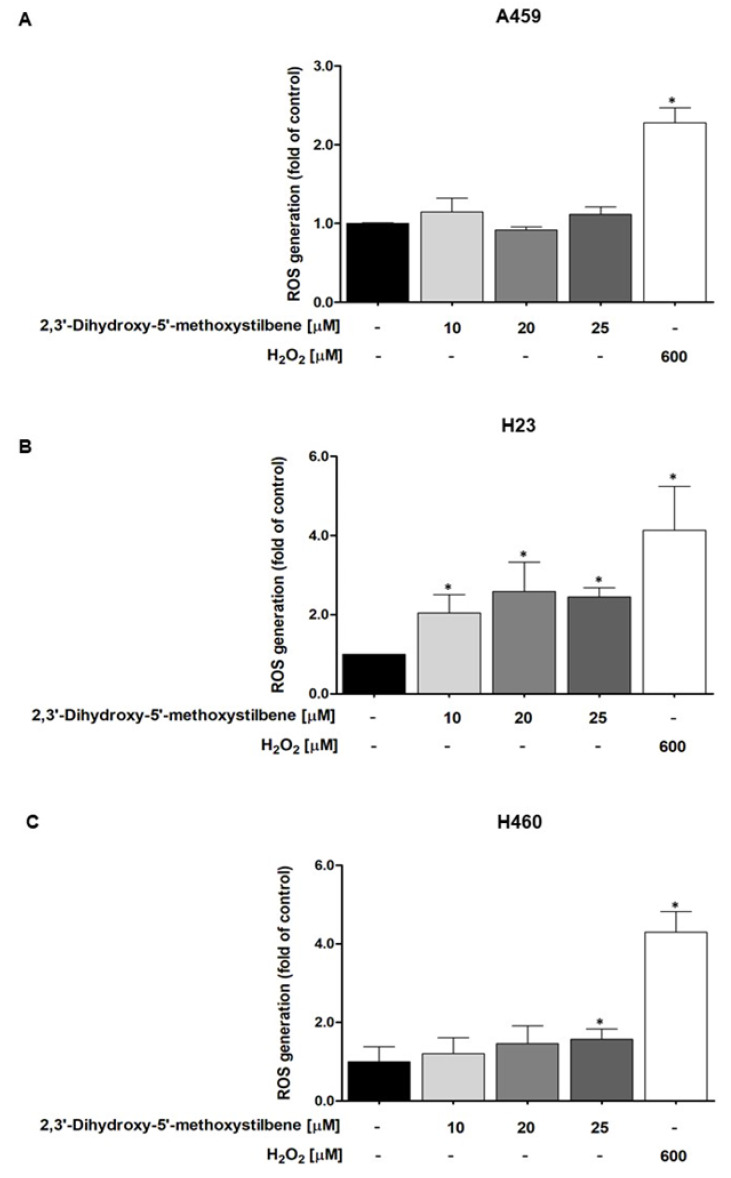
Induction of oxidative stress by 2,3′-dihydroxy-5′-methoxystilbene in NSCLC cell lines with differential antioxidant capacity. Reactive oxygen species (ROS) generation was measured using the DCFH-DA fluorescence assay in (**A**) A549, (**B**) H23, and (**C**) H460 cells treated with increasing concentrations (10, 20, and 25 μM) of 2,3′-dihydroxy-5′-methoxystilbene for 1 h. Hydrogen peroxide (H_2_O_2_, 600 μM) was used as a positive control. Data are expressed as fold change relative to the untreated control and are presented as the mean ± SEM (*n* = 3). Statistical significance was determined using a *t*-test (* *p* < 0.05).

**Figure 7:**
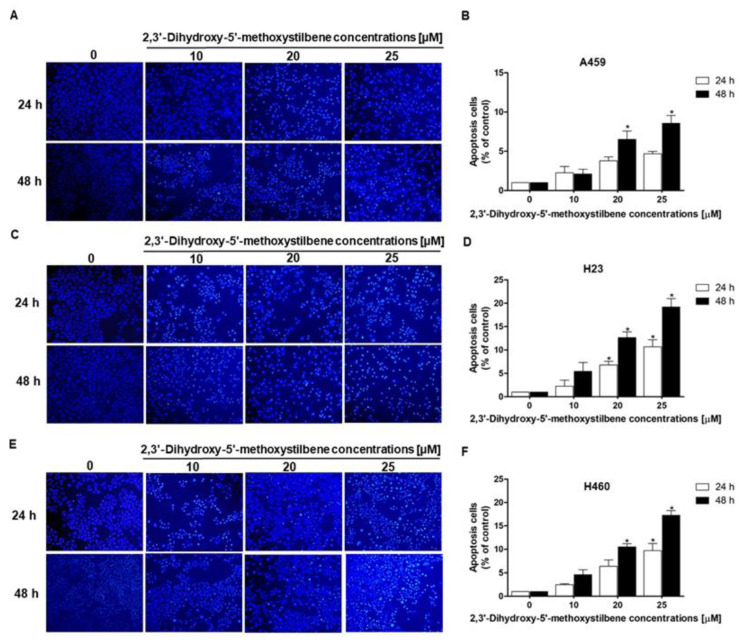
Apoptosis induction by 2,3′-dihydroxy-5′-methoxystilbene in NSCLC cell lines. Representative fluorescence microscopy images of Hoechst 33342 nuclear staining showing apoptotic cell morphology in (**A**) A549, (**C**) H23, and (**E**) H460 cells treated with increasing concentrations (0, 10, 20, and 25 μM) of 2,3′-dihydroxy-5′-methoxystilbene for 24 and 48 h. Apoptotic cells are characterized by nuclear condensation, chromatin fragmentation, and formation of apoptotic bodies. Quantitative analysis of apoptotic cell populations expressed as a percentage of the control for (**B**) A549, (**D**) H23, and (**F**) H460 cells at 24 h and 48 h. Data are presented as the mean ± SEM (*n* = 3). Statistical significance was determined using a *t*-test (* *p* < 0.05).

Differential expression of the Nrf2/HO-1 antioxidant axis contributes to distinct redox homeostasis thresholds in NSCLC subtypes. To assess whether oxidative stress was responsible for the selective cytotoxic effects of 2,3′-dihydroxy-5′-methoxystilbene, we measured the production of reactive oxygen species (ROS) using a DCFH-DA fluorescence assay. All cell lines were incubated with increasing concentrations of the tested compounds (10–25 μM) for 1 h (Figure 6). H23 cells exhibited the most pronounced susceptibility to oxidative stress, displaying statistically significant ROS elevation at all tested concentrations, with the relative fluorescence intensity increasing by approximately 2.0-, 2.5-, and 2.4-fold over controls at 10, 20, and 25 μM, respectively (Figure 7B; *p* < 0.05). The increase in ROS production suggests that the antioxidant defense system in H23 cells was compromised. In contrast, H460 cells showed moderate sensitivity, with a notable increase in ROS production only at the highest concentration (25 μM; 1.7-fold increase, *p* < 0.05), whereas lower concentrations (10 and 20 μM) resulted in non-significant increases in ROS production (Figure 6C). In contrast, A549 cells maintained robust redox homeostasis, showing no significant ROS accumulation at any concentration (Figure 6A). These findings suggest that the selective cytotoxicity of 2,3′-dihydroxy-5′-methoxystilbene is closely associated with the varying antioxidant capacities of the cell lines, potentially influenced by differences in Nrf2/HO-1 activity.

### 2.6. Effect of 2,3′-Dihydroxy-5′-Methoxystilbene on Apoptosis in NSCLC Cells

Programmed cell death plays a role in eliminating malignant cells and is a primary target of anticancer therapeutic strategies. To determine whether apoptosis underlies the antiproliferative effects of 2,3′-dihydroxy-5′-methoxystilbene, we evaluated morphological changes in the nuclei of NSCLC cell lines using Hoechst 33342 staining. Following treatment with increasing concentrations (10–25 μM) for 24 and 48 h, the treated cells exhibited characteristic features of apoptosis, including nuclear condensation and chromatin fragmentation, in contrast to the intact nuclei observed in the controls (Figure 7A,C,E). Quantitative assessment revealed differential sensitivity between the cell lines tested. H23 cells displayed the most pronounced apoptotic response in a dose-dependent manner, with apoptotic rates reaching approximately 5%, 12%, and 19% at 10, 20, and 25 μM, respectively, after 48 h of treatment (Figure 7D). H460 cells exhibited intermediate sensitivity, with apoptotic populations increasing from approximately 4% to 17% across the same concentration range (Figure 7F). Conversely, A549 cells demonstrated the lowest susceptibility, with modest increases in apoptosis of approximately 2%, 6%, and 8% at 10, 20, and 25 μM, respectively (Figure 7B). These findings suggest that apoptosis induction significantly contributes to the cytotoxic efficacy of 2,3′-dihydroxy-5′-methoxystilbene.

### 2.7. Computational Analysis of 2,3′-Dihydroxy-5′-Methoxystilbene: Target Identification and Molecular Mechanisms in NSCLC

#### 2.7.1. Target Network Analysis and Protein–Protein Interaction Mapping

**Figure 8:**
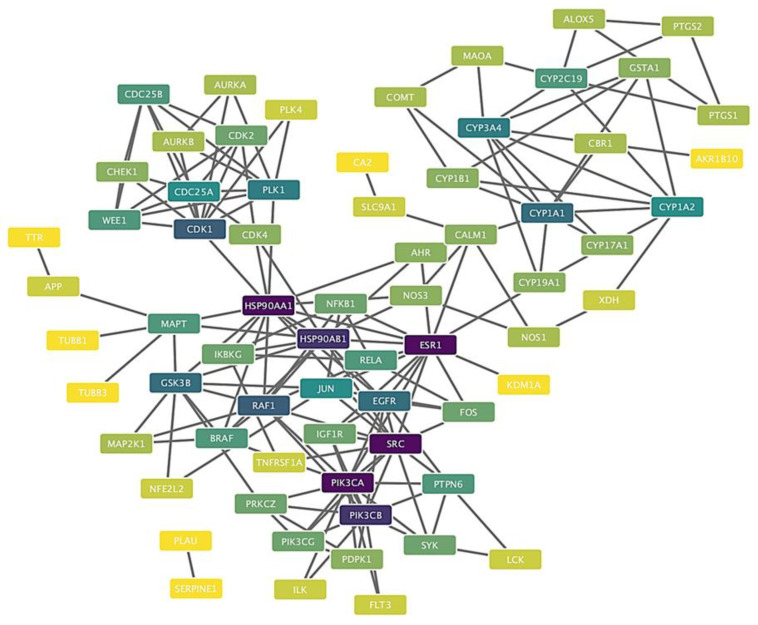
Protein–protein interaction network highlighting central cancer-related signaling proteins. A network diagram showing protein–protein interactions among signaling molecules relevant to cancer biology and drug discovery. Purple nodes highlight central hubs (HSP90AA1, HSP90AB1, ESR1, SRC, PIK3CA, PIK3CB), representing proteins implicated in cell signaling, on-cogenesis, and therapeutic targeting. Green/Teal nodes denote intermediate connectivity, involved in signal modulation and cellular effects. Yellow nodes indicate peripheral proteins with fewer interactions, associated with specialized functions.

To elucidate the molecular interactome of 2,3′-dihydroxy-5′-methoxystilbene, we performed computational target prediction. Venn diagram analysis identified 101 intersecting targets between the compound and NSCLC-associated genes (1.1% intersection), suggesting a narrow therapeutic window (Figure 8, Table S1). Subsequent protein–protein interaction (PPI) network analysis revealed a hierarchical topology characterized by interconnected functional clusters rather than isolated interactions. Topological analysis identified central regulatory nodes that serve as convergence points for oncogenic signaling pathways. Notably, the critical hub proteins, including HSP90AA1, HSP90AB1, ESR1, SRC, and PIK3CA, exhibited extensive connectivity with multiple nodes. The identification of PIK3CA (the catalytic subunit of PI3K) as the primary hub provides strong computational support for the experimental modulation of the PI3K/AKT signaling axis. Visualized through degree-based mapping, these results indicate that 2,3′-dihydroxy-5′-methoxystilbene may exert pleiotropic anticancer effects by simultaneously targeting key regulatory kinases and transcription factors.

#### 2.7.2. Gene Ontology (GO) and KEGG Pathway Enrichment Analysis of 2,3′-Dihydroxy-5′-Methoxystilbene-Associated Targets

**Figure 9:**
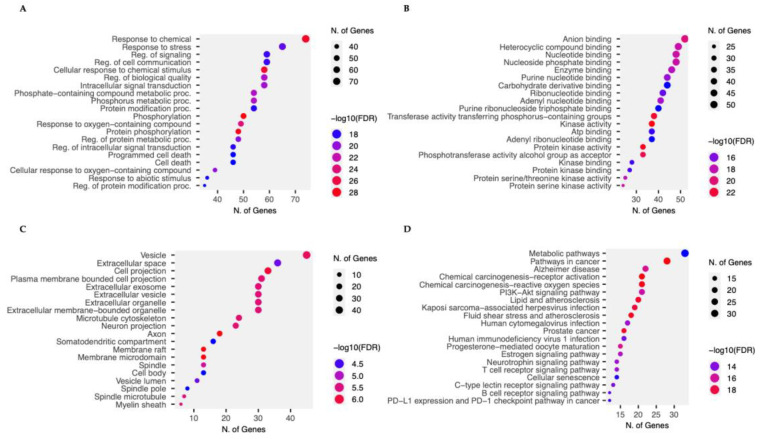
Gene Ontology (GO) and KEGG pathway enrichment analysis of 2,3′-dihydroxy-5′-methoxystilbene-targeted genes. (**A**) Biological Process (BP): Top enriched GO terms related to cell death, chemical response, the regulation of signaling, and apoptosis. (**B**) Molecular Function (MF): Enrichment in catalytic activity, ATP binding, protein kinase activity, and nucleotide binding. (**C**) Cellular Component (CC): Targeted proteins are predominantly associated with intracellular anatomical structures, membranes, and cytoplasmic components. (**D**) KEGG Pathway Analysis: Major enriched pathways include cancer-related signaling pathways, such as PI3K/Akt, pathways in cancer, chemical carcinogenesis receptor activation, and chemical carcinogenesis reactive oxygen species signaling. Gene counts reflect the number of target genes associated with each GO term or KEGG pathway. Data were analyzed using enrichment tools with a significance threshold of *p* < 0.05.

Gene Ontology (GO) analysis elucidated the biological functions of the identified targets. In the Biological Process (BP) category (Figure 9, Table S1), the targets were significantly enriched in cellular metabolic processes (>80 genes) and response to chemical stimuli (60 genes), suggesting a fundamental disruption of cancer cell bioenergetics. Notably, the enrichment of cell death and apoptotic regulatory pathways provides mechanistic support for the pro-apoptotic activity observed in in vitro studies. In terms of molecular function (MF), the analysis demonstrated a predominant association with catalytic and protein kinase activities (140 genes), as well as ATP- and ion-binding functions. This profile supports a kinase-centric mechanism of action, which is consistent with the hypothesized modulation of AKT signaling. Cellular Component (CC) analysis revealed that the targets are extensively distributed across the cytoplasm (100 genes), membrane-bound organelles, and nucleus, implying multi-compartmental regulation of cell proliferation and metabolism. Crucially, Kyoto Encyclopedia of Genes and Genomes (KEGG) pathway enrichment analysis identified the PI3K/AKT signaling pathway (25 genes) as the principal target (Figure 9). The concurrent enrichment of general cancer and metabolic pathways underscores the pleiotropic potential of this compound to target critical regulatory nodes in kinase signaling and cellular metabolism. These enrichment data are exploratory and were used to prioritize candidate pathways and targets for future functional validation, rather than to claim that each enriched gene is directly modulated by 2,3′-dihydroxy-5′-methoxystilbene under the current experimental conditions.

#### 2.7.3. Computational Modeling of AKT with Molecular Docking and Structural Validation

**Figure 10:**
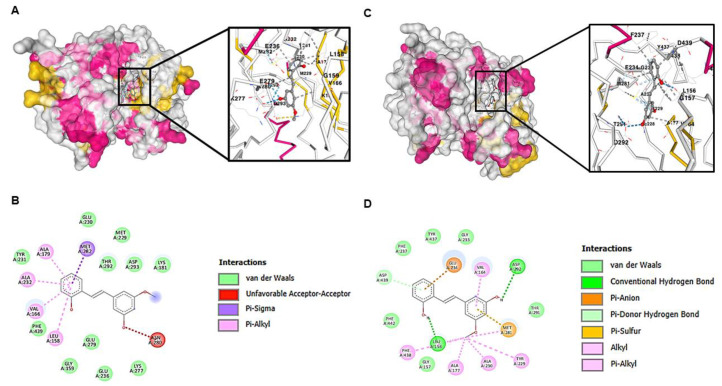
Molecular docking analysis of 2,3′-dihydroxy-5′-methoxystilbene with activated and non-activated AKT conformations. (**A**) Surface representation of activated AKT showing 2,3′-dihydroxy-5′-methoxystilbene binding within the ATP-binding domain. The zoomed-in view displays the compound positioned in the C1 pocket with detailed molecular contacts. (**B**) Two-dimensional interaction diagram of 2,3′-dihydroxy-5′-methoxystilbene with activated AKT. (**C**) Surface representation of non-activated AKT demonstrating compound binding within the ATP-binding region with altered binding pocket geometry compared to the activated form. (**D**) Two-dimensional interaction map of 2,3′-dihydroxy-5′-methoxystilbene with non-activated AKT. Binding energies (Vina scores): −7.8 kcal/mol for non-activated Akt (3MVH, pocket C1) and −7.7 kcal/mol for activated Akt (1O6L, pocket C1).

Direct protein–protein interactions represent critical regulatory mechanisms in cancer cell signaling, with PI3K/AKT serving as a central hub for oncogenic pathway activation. Next, we investigated whether 2,3′-dihydroxy-5′-methoxystilbene exhibits differential binding affinity for AKT conformational states through molecular docking analysis to elucidate its selective targeting mechanism. Molecular docking analysis revealed distinct interaction profiles between the activated and non-activated AKT conformational states (Figure 10A,C). Molecular docking using CB-Dock2 identified the largest pocket (C1) of AKT as the preferred binding site for 2,3′-dihydroxy-5′-methoxystilbene in both conformational states. For the non-activated AKT structure (PDB: 3MVH), docking to pocket C1 yielded a Vina score of −7.8 kcal/mol, while docking to the corresponding pocket in activated AKT (PDB: 1O6L) produced a Vina score of −7.7 kcal/mol. These negative binding energies are consistent with moderately strong affinity in the low-micromolar range and support the formation of stable complexes within the ATP-binding region as characterized above. Regarding these preferential binding characteristics, activated AKT binding (Figure 10B) exhibited extensive van der Waals contacts with multiple residues (GLU230, MET229, ASP293, LYS181, THR292, GLU279, GLU236, ALA179, ALA232, GLY159, LEU158, and PHE439), π-σ interactions with MET282, and π–alkyl interactions with hydrophobic residues, creating a tightly packed, hydrophobic-dominated binding interface with optimal shape complementarity and high binding affinity. Non-activated AKT demonstrated reduced van der Waals contact density with compensatory polar interactions, including conventional hydrogen bonding with ASP292 and LEU156, π–anion and π–donor hydrogen bonds, and π–sulfur interactions with GLU234 and MET281, resulting in a more diffuse binding interface with decreased optimization, as shown in Figure 10D. These differential interaction profiles indicate that 2,3′-dihydroxy-5′-methoxystilbene functions as a conformationally selective AKT inhibitor, preferentially targeting the activated state through optimized hydrophobic interactions while maintaining a reduced affinity for basal AKT signaling. This finding provides molecular validation for the selective anticancer activity against malignant cells with a hyper-activated AKT pathway.

### 2.8. The Effect of 2,3′-Dihydroxy-5′-Methoxystilbene Inhibition on the Akt/GSK3β Signaling Pathway

**Figure 11:**
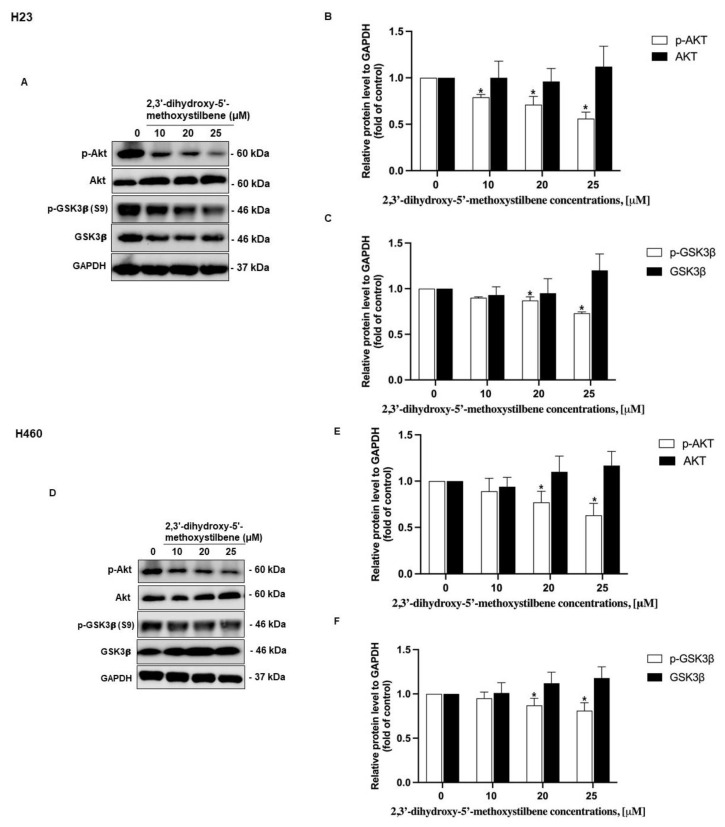
Western blot analysis of 2,3′-dihydroxy-5′-methoxystilbene’s effects on Akt and GSK3β phosphorylation and total protein levels in H23 and H460 NSCLC cells. (**A**,**D**) Representative immunoblots for phosphorylated Akt (p-Akt), total Akt, phosphorylated GSK3β (p-GSK3β), total GSK3β, and GAPDH after treatment with increasing concentrations of 2,3′-dihydroxy-5′-methoxystilbene in H23 and H460 cells, respectively. (**B**,**C**) Densitometric analyses of p-Akt and p-GSK3β normalized to total Akt and GSK3β, respectively, as well as total Akt and GSK3β normalized to GAPDH, in H23 cells. (**E**,**F**) Densitometric analyses of p-Akt and p-GSK3β normalized to total Akt and GSK3β, respectively, as well as total Akt and GSK3β normalized to GAPDH, in H460 cells. Data are shown as mean ± SD from at least three independent experiments. * *p* < 0.05 vs. control.

Computational target identification revealed a selective interaction profile between 2,3′-dihydroxy-5′-methoxystilbene and the PI3K/AKT pathway, with predicted interactions at the AKT active site in both active and inactive conformations of the latter. To evaluate the downstream consequences of these interactions, Western blotting and densitometric analyses were performed in H23 and H460 NSCLC cell lines. As shown in Figure 11A,F, exposure to increasing concentrations of 2,3′-dihydroxy-5′-methoxystilbene (0, 10, 20, and 25 μM) resulted in a dose-dependent decrease in the phosphorylation of Akt (p-Akt) and its downstream target, GSK3β (p-GSK3β), in both cell types. Quantitatively, in H23 cells (Figure 11B), p-Akt (normalized to GAPDH) decreased from 1.0 (control) to 0.85 (10 μM), 0.80 (20 μM), and 0.70 (25 μM), whereas p-GSK3β (Figure 11C) decreased from 1.0 to 0.95, 0.85, and 0.75, respectively. Similar effects were observed in H460 cells (Figure 11E); p-Akt decreased from 1.0 to 0.85, 0.80, and 0.65, and (Figure 11F) p-GSK3β decreased from 1.0 to 0.90, 0.85, and 0.70 with increasing concentrations. Total Akt and GSK3β protein levels remained stable throughout the treatment (approximately 0.95–1.1-fold of control). These findings suggest that 2,3′-dihydroxy-5′-methoxystilbene may inhibit the kinase activity of activated AKT, subsequently reducing the phosphorylation of GSK3β without altering the total expression levels of these proteins. This modulation of phosphorylation may reflect the direct inhibition of AKT enzymatic activity or interference with upstream kinases or pathway crosstalk, rather than global changes in protein expression levels (Figure 11). These data indicate that 2,3′-dihydroxy-5′-methoxystilbene attenuates AKT and GSK3β activation; however, additional loss-of-function experiments (e.g., AKT/GSK3β knockdown or pharmacological inhibition) will be required to establish whether this pathway is causally linked to the observed antiproliferative and antimigratory effects.

### 2.9. 2,3′- Dihydroxy-5′-Methoxystilbene Enhances Cisplatin-Induced Cytotoxicity in NSCLC Cells

**Figure 12:**
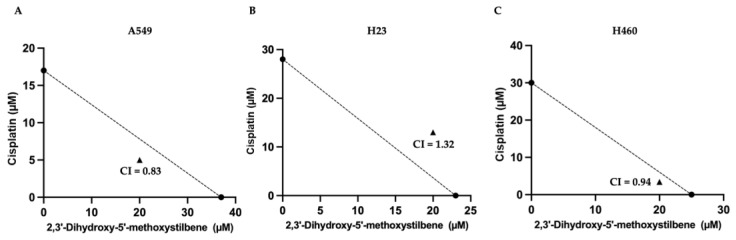
Isobologram graphs of the combined effects of 2,3′-dihydroxy-5′-methoxystilbene and cisplatin on A549 (**A**), H23 (**B**), and H460 (**C**) cells after 48 h. co-treatment. Combination index (CI) values indicate synergism in A549 (CI = 0.83), antagonism in H23 (CI = 1.32), and mild synergism in H460 (CI = 0.94). The CI values suggested the following: antagonism when CI > 1, additive interaction when CI = 1, and synergism when CI < 0.1. Data represent three independent experiments.

Chemoresistance and dose-limiting toxicity are major clinical hurdles in cisplatin-based chemotherapy for non-small-cell lung cancer (NSCLC). To determine whether 2,3′-dihydroxy-5′-methoxystilbene functions as a chemosensitizer, we evaluated the cytotoxic effects of cisplatin (5–50 μM) combined with a fixed concentration of stilbene (20 μM) for 48 h. Isobolographic analysis revealed distinct cell type-specific interaction profiles (Figure 12). In A549 cells, the combination exerted a synergistic effect (CI = 0.83), reducing the effective IC_50_ of cisplatin to 5 μM in the presence of the compound (Figure 12A). Similarly, H460 cells displayed favorable interactions, ranging from additive to mildly synergistic (CI = 0.94), with a combination IC_50_ of 3.4 μM for cisplatin (Figure 12C). In contrast, H23 cells exhibited antagonism (CI = 1.32) and required a higher concentration of cisplatin (~13 μM) to achieve the same effect (Figure 12B). Collectively, these findings demonstrate that 2,3′-dihydroxy-5′-methoxystilbene possesses a dose-sparing potential for cisplatin in A549 and H460 cells, although the interaction is contingent on the specific genetic background of the cell line.

## 3. Discussion

This study investigated the anticancer activity of 2,3′-dihydroxy-5′-methoxystilbene, a naturally occurring stilbene derivative, in three NSCLC cell lines (A549, H23, and H460), and compared it to normal NIH/3T3 fibroblasts. The compound was evaluated for multiple cancer hallmarks relevant to NSCLC. Specifically, we examined its ability to suppress proliferative signaling, induce apoptosis, inhibit clonogenic survival (resisting cell death/replicative immortality), reduce migratory capacity (invasion and metastasis), disrupt redox homeostasis (deregulated metabolism/oxidative stress), and modulate key oncogenic pathways in cancer cells. Furthermore, computational modeling was performed to predict molecular targets and identify mechanistic hubs associated with its anticancer activity, including the PI3K/AKT/GSK3β signaling pathway. In addition to evaluating the effects of 2,3′-dihydroxy-5′-methoxystilbene alone, combination studies with cisplatin were performed to explore potential synergistic or additive anticancer effects [22]. In the present study, 2,3′-dihydroxy-5′-methoxystilbene exhibited selective cytotoxicity against NSCLC cell lines, with the most pronounced effects observed in H23 and H460 cells (IC_50_: ~23 to 24 μM) compared to A549 cells (IC_50_: 37 μM). Crucially, normal NIH/3T3 fibroblasts displayed limited sensitivity to the compound (IC_50_ > 100 μM at 48 h). In contrast, although cisplatin achieved lower IC_50_ values in cancer cells, it exhibited substantial toxicity toward normal fibroblasts, underscoring the superior selectivity profile of stilbene derivatives [22]. In contrast, Reflexanbene-F, a structurally modified stilbene featuring both a methoxy group and a bulky p-menthene substituent, demonstrated a significantly greater antiproliferative effect in A549 cells (IC_50_ = 5.09 μM at 48 h). This comparison highlights that increased hydrophobicity and steric complexity from additional substituents, as in Reflexanbene F, can markedly enhance anticancer activity beyond natural analogs, as shown by Fu et al., 2022 [23]. In comparison to tri-hydroxylated resveratrol, 2,3′-dihydroxy-5′-methoxystilbene exhibits greater molar potency, with IC_50_ values ranging from 23 to 37 µM, in contrast to resveratrol, which demonstrates IC_50_ values of 87 to 218 µM for inducing significant cytotoxicity according to Balasubramani et al., 2019 [24]. This approximately 2- to 4-fold increase in potency is likely attributable to the presence of a single 5′-methoxy substituent, which enhances lipophilicity and membrane permeability compared to the hydrophilic tri-hydroxylated resveratrol scaffold [24]. As previously reported, cis-configured stilbenes, such as combretastatin A-4 (CA-4) and its fatty acid conjugates, exhibit potent microtubule-targeting activity, with IC_50_ values in the nanomolar range (approximately 0.01–0.06 µM) against NCI-H460 cells. These findings underscore the pivotal role of cis geometry in facilitating optimal tubulin-binding interactions [25]. Furthermore, the hybridization of the trans-stilbene scaffold with a tetrahydroisoquinoline heterocycle significantly enhanced cytotoxic potency. For instance, hybrid compound 16e exhibited nanomolar efficacy against A549 cells (IC_50_ ≈ 25 nM), surpassing the potency of colchicine. Mechanistic studies revealed that the introduction of electron-withdrawing substituents and the tetrahydroisoquinoline moiety converts the moderately active stilbene scaffold into a highly potent cytotoxin that functions by inhibiting tubulin polymerization, inducing G2/M cell cycle arrest, and triggering mitochondrial-dependent apoptosis [26]. Conversely, their trans-analogs often exhibit IC_50_ values in the micromolar range (2.4 to 4.9 µM), highlighting a 3- to 4-fold potency gap attributable to the specific binding requirements of the tubulin colchicine site [25,27]. Collectively, these data indicate that 2,3′-dihydroxy-5′-methoxystilbene functions as a selective lead compound, particularly for the H23 and H460 subtypes. Although it lacks the nanomolar potency of tubulin-targeting cis-stilbenes or complex hybrids [28], its trans-geometry offers a stability advantage and a distinct, likely, kinase-mediated mechanism of action. When comparing the structure of 2,3′-dihydroxy-5′-methoxystilbene with resveratrol (3,4′,5-trihydroxy-trans-stilbene) and pterostilbene (3,5-dimethoxy-4′-hydroxystilbene), resveratrol typically shows IC_50_ values in the mid-to-high tens of micromolar against many solid tumor cell lines after 48 to 72 h of exposure, whereas pterostilbene demonstrates low micromolar IC_50_ values under comparable conditions, reflecting increased lipophilicity and metabolic stability associated with A-ring dimethoxylation. In our NSCLC models under uniform 48 h MTT assay conditions, 2,3′-dihydroxy-5′-methoxystilbene, which contains two hydroxyl groups and a single methoxy group on ring B, achieved IC_50_ values of approximately 24 µM in H23 and H460 cells, with substantially weaker effects in A549 cells and low toxicity in NIH/3T3 fibroblasts (IC_50_ > 100 µM). These quantitative differences support the broader stilbene SAR principle that increasing the extent of methoxylation tends to lower IC_50_ values (i.e., higher antiproliferative potency), whereas the specific number and position of methoxy and hydroxy groups modulate target selectivity and safety [29]. Although the relative lack of toxicity in NIH/3T3 cells suggests that 2,3′-dihydroxy-5′-methoxystilbene is not broadly cytotoxic, the use of murine fibroblasts as a non-malignant comparator rather than human lung epithelium represents a limitation. These findings cannot be directly extrapolated to human lung tissue; consequently, future studies should employ non-transformed human lung epithelial models (e.g., BEAS2B) to define the therapeutic window more precisely. Future optimization strategies for resistant lines, such as A549, may require scaffold modifications, such as additional methoxylation, cis-constraint, or heterocycle fusion, to achieve submicromolar efficacy without compromising the compound’s favorable selectivity.

Colony formation and migration capacity are critical surrogates for tumor recurrence and metastatic potential. In this study, 2,3′-dihydroxy-5′-methoxystilbene exhibited cell line-dependent inhibition of long-term reproductive potential. H23 cells exhibited the highest sensitivity, displaying significant dose-dependent reductions in both colony number and size at 10, 20, and 25 μM. H460 cells showed moderate inhibition at higher doses, whereas A549 cells were relatively resistant, with significant suppression observed only at 25 μM, primarily affecting the colony size. Regarding migration, the compound induced a distinct response profile, with H460 cells exhibiting near-complete migration arrest at 25 μM, whereas A549 cells displayed intermediate, dose-dependent inhibition. Interestingly, H23 cells, despite being highly sensitive to clonogenic inhibition, showed only a modest reduction in migration. This divergence suggests that the compound targets distinct molecular drivers of proliferation and motility in different genetic backgrounds. Comparative analysis with structurally related methoxylated stilbenes revealed consistent anti-metastatic trends. For instance, trimethoxystilbene (TMS) inhibits A549 colony formation in a dose-dependent manner (IC_50_:8.6 μM) and significantly impairs migration at a concentration of 8 μM. Similarly, 3,5,4′-trimethoxy-trans-stilbene (MR-3) robustly suppresses adhesion, migration, and invasion at non-cytotoxic concentrations (5 μM) by downregulating MMP-2 via the JNK/p38 MAPK and NF-κB/AP-1 signaling pathways [22,30]. Although 2,3′-dihydroxy-5′-methoxystilbene exhibits slightly lower potency than fully methylated analogs, it maintains a favorable selectivity profile, effectively targeting aggressive NSCLC phenotypes while sparing normal fibroblasts [26]. Collectively, these findings support the therapeutic potential of 2,3′-dihydroxy-5′-methoxystilbene in mitigating NSCLC recurrence and dissemination, warranting further mechanistic investigations into its effects on matrix metalloproteinase (MMP) regulation and cytoskeletal dynamics in NSCLC cells.

Inducing oxidative stress is a pivotal strategy for targeted cancer therapy, which exploits the altered redox thresholds inherent to malignant phenotypes. The present study revealed a distinct inverse correlation between basal antioxidant capacity and susceptibility to 2,3′-dihydroxy-5′-methoxystilbene-induced oxidative stress. H23 cells, characterized by compromised Nrf2/HO-1 defense mechanisms [31], exhibited significant, dose-dependent increases in ROS at all concentrations tested, reaching up to 2.5-fold above baseline, suggesting a robust oxidative stress response due to compromised antioxidant defense mechanisms. H460 cells showed moderate susceptibility, with significant ROS elevation only at the highest dose, indicating partial resilience or intermediate Nrf2/HO-1 functions. In contrast, A549 cells displayed strong resistance to ROS accumulation, with only minimal increases at any concentration, consistent with highly active antioxidant systems likely mediated by upregulation of Nrf2/HO-1. In our panel, H23 and H460 cells consistently exhibited higher sensitivity to 2,3′-dihydroxy-5′-methoxystilbene than A549 cells, which can be explained by differences in redox balance and signaling dependency. H23 cells showed the most pronounced ROS accumulation and apoptotic response, whereas H460 cells displayed intermediate ROS and apoptosis, indicating that both cell lines possessed relatively limited antioxidant reserves and were more vulnerable to the combined effects of oxidative stress and AKT/GSK3β inhibition. In contrast, A549 cells maintained redox homeostasis with no significant increase in ROS and only modest apoptosis under the same conditions, suggesting a more robust Nrf2/HO-1-driven antioxidant capacity and reduced reliance on AKT signaling, which likely underlies their relative resistance to this combination of compounds. These results are consistent with those of previous investigations of other stilbene derivatives, where specific structural and functional groups critically influence pro-oxidant capacity. For example, trimethoxy-substituted or brominated stilbenes, owing to their enhanced lipophilicity and electron-donating groups, have been reported to generate ROS more effectively in NSCLC cell lines, particularly in those with lower baseline antioxidant protection [22,32]. In the case of A549 cells, studies on both methoxylated and basic stilbenes have shown that their oxidant-driving effects are often blunted, reinforcing the notion that antioxidant machinery, modulated by Nrf2/HO-1 status, dictates the cellular response [26,30]. Collectively, these data demonstrate that 2,3′-dihydroxy-5′-methoxystilbene functions as a “redox-directed” agent, selectively eliminating NSCLC cells with impaired antioxidant machinery (H23/H460) while sparing those with robust Nrf2-mediated defense (A549 cells).

Apoptosis induction is a cornerstone of anticancer therapy and a critical mechanism for overcoming tumor progression and treatment resistance. In the present study, morphological assessment using Hoechst 33342 staining demonstrated that 2,3′-dihydroxy-5′-methoxystilbene induced apoptosis in a cell line-dependent manner. H23 cells exhibited the most robust response, with apoptotic populations reaching approximately 19% at 25 μM (48 h), whereas H460 cells displayed intermediate sensitivity (17%). In contrast, A549 cells were the least responsive, with apoptosis peaking at 8%. This differential sensitivity likely reflects the capacity of the compound to exploit specific vulnerabilities in redox homeostasis and the dependence on signaling pathways. A comparative analysis with the existing literature highlighted the critical role of structural elements in determining pro-apoptotic potency. Methoxy- and hydroxy-substituted derivatives, particularly those with ortho- or para-substitutions, often exhibit enhanced apoptotic efficacy. For instance, trimethoxystilbene (TMS) has been shown to robustly activate caspase-3 and induce nuclear fragmentation in lung cancer cell lines [22]. Furthermore, halogenated or cyclized analogs, such as combretastatin derivatives, amplify these effects by promoting mitochondrial dysfunction and p53 pathway activation, thereby yielding superior apoptotic rates [28,32]. Conversely, unsubstituted or mono-hydroxylated stilbenes often exhibit attenuated activities, necessitating higher concentrations to achieve comparable effects. These findings underscore the importance of strategically modifying functional groups to optimize the pro-apoptotic pharmacophore of stilbene derivatives. It should be noted that this study relied on nuclear morphology (Hoechst 33342 staining) to assess cell death, which may have underestimated early or non-classical apoptotic events. Future investigations should incorporate multiparametric flow cytometry (e.g., Annexin V/PI staining) and caspase activity assays to provide quantitative validation. Additionally, elucidating the precise interplay between mitochondrial dynamics and death receptor signaling is necessary to fully characterize the apoptotic cascade. Exploring synergistic combinations with other redox-modulating agents could further enhance the anticancer selectivity.

Stilbenes, including resveratrol and its optimized analogs, modulate oncogenic survival pathways and exert multifaceted antitumor effects in lung cancer models. Incorporating recent evidence into this discussion enhances the mechanistic understanding of the interaction between 2,3′-dihydroxy-5′-methoxystilbene and the PI3K/AKT/GSK3β axis. This aligns with a broader body of research demonstrating the suppression of proliferation, induction of apoptosis, inhibition of colony formation, and reduction in migration and invasion through kinase- and redox-centered mechanisms [22,30,33]. Computational mapping and docking studies predicted a selective target profile for 2,3′-dihydroxy-5′-methoxystilbene, revealing a 1.1% overlap across 101 NSCLC-associated proteins and clustering at high-degree hubs (HSP90AA1, HSP90AB1, ESR1, SRC, and PIK3CA) that are central to PI3K/AKT signaling and adaptive stress responses [34,35]. Gene Ontology (GO) and KEGG enrichment analyses highlighted catalytic and kinase activities, regulation of apoptosis, metabolic processes, and explicit representation of the PI3K/AKT pathway. Docking studies favored activated AKT through dense hydrophobic contacts, supporting conformationally selective inhibition, which is consistent with the tumor-biased activity [35]. The concordance with previous stilbene studies further substantiates this proposed mechanism. Pinostilbene was found to suppress PI3K/AKT signaling and epithelial–mesenchymal transition (EMT) in A549 cells, with reduced migration in functional assays, aligning with 2,3′-dihydroxy-5′-methoxystilbene’s antimigratory profile and implicating the same pathway [36]. Similarly, 3,5,4′-trimethoxy-trans stilbene (MR-3) attenuates adhesion, migration, and invasion by downregulating MMP-2 and suppressing the JNK/p38 MAPK pathways, which are interconnected with the PI3K/AKT pathway in controlling motility and survival [30]. Resveratrol and its analogs have been shown to reduce p-AKT/p-GSK3β, arrest the cell cycle, and promote apoptosis via intrinsic and extrinsic pathways, mirroring the pro-apoptotic and anti-clonogenic actions of 2,3′-dihydroxy-5′-methoxystilbene [37,38]. Furthermore, 3,4,4′-trihydroxy-trans-stilbene induced apoptosis and autophagy in A549 cells via mTOR inhibition, consistent with downstream PI3K/AKT pathway blockade [38]. Additionally, 4,4′-dihydroxy-transstilbenes reduced stemness, clonogenicity, and spheroid formation in A549 cells, functionally overlapping with PI3K/AKT-dependent survival and self-renewal pathways, even when direct readouts were not measured [39]. Collectively, these data converge on a coherent model in which 2,3’-dihydroxy-5’-methoxystilbene and related stilbenes operate through kinase-centric networks dominated by PI3K/AKT/GSK3β and interconnected MAPK/mTOR modules to induce apoptosis, inhibit EMT and migration, and reduce clonogenic potential [16,22,40,41]. Although the coordinated decrease in p-AKT and p-GSK3β, along with reduced proliferation, clonogenicity, and migration, is consistent with an AKT/GSK3β-mediated mechanism, these observations remain correlative. The present study did not utilize siRNA-mediated knockdown or selective pharmacological inhibition of AKT and GSK3β to functionally validate this pathway. Future experiments will address this limitation by determining whether targeting this axis recapitulates or is necessary for the antitumor and anti-invasive effects of 2,3′-dihydroxy-5′-methoxystilbene. Structural features, particularly methoxy/hydroxy substitution patterns, enhance potency and selectivity by improving target engagement and modulating redox stress, thereby explaining the cell line-specific sensitivity and reduced off-target toxicity [33]. However, this study has some limitations, which can be addressed in future research. Computational predictions may not fully capture microenvironmental complexity, post-translational states, or system feedback; thus, rigorous validation with phosphoproteomics, multiomics, time-resolved signaling assays, and in vivo efficacy/toxicity studies is required to confirm their accuracy. Mechanistic dissection of AKT conformational selectivity, biomarker development for PI3K/AKT dependency, and combination testing with PI3K/mTOR or MAPK inhibitors could refine therapeutic windows and reveal biomarker-guided strategies [34,35]. In summary, the integration of these references reinforces that the predicted and observed activities of 2,3′-dihydroxy-5′-methoxystilbene are mechanistically aligned with PI3K/AKT/GSK3β inhibition and its downstream regulation of proliferation, apoptosis, clonogenicity, and migration in non-small-cell lung cancer (NSCLC), consistent with the broader stilbene literature [30,36,38].

To elucidate the molecular mechanism underlying the observed antiproliferative and pro-apoptotic effects, we investigated the modulation of the PI3K/AKT signaling cascade. Western blot analysis revealed that treatment with 2,3′-dihydroxy-5′-methoxystilbene significantly reduced the phosphorylation levels of AKT (p-AKT) and its downstream effector GSK3β (p-GSK3β) in both H23 and H460 cells, whereas total protein levels remained unaltered. This suggests that the compound functions by suppressing kinase activation rather than by inducing protein degradation. Notably, the binding energies obtained (−7.8 and −7.7 kcal/mol for non-activated and activated AKT, respectively) and the dominance of hydrophobic contacts with limited hydrogen bonding are fully compatible with the physicochemical profile of 2,3′-dihydroxy-5′-methoxystilbene and with the micromolar concentrations required to experimentally reduce p-AKT/p-GSK3β levels and inhibit NSCLC cell viability, indicating good agreement between in silico predictions and observed biological activity. These findings are consistent with reports on structurally related stilbenes. For instance, pinostilbene has been shown to delay pulmonary fibrosis and inhibit epithelial–mesenchymal transition (EMT) by directly modulating the PI3K/AKT pathway [26]. Similarly, δ-viniferin-induced apoptosis in NSCLC cells is mechanistically linked to a reduction in phosphorylated AKT, confirming pathway interference [42]. Taken together, the decrease in p-AKT and p-GSK3β, combined with selective apoptosis and ROS induction, supports a model in which 2,3′-dihydroxy-5′-methoxystilbene attenuates AKT signaling and perturbs redox homeostasis in susceptible NSCLC cells. Nonetheless, our current study only investigated a limited number of nodes within this pathway and did not establish the causal roles of individual GO/KEGG-derived targets. These network-based targets therefore represent testable hypotheses that will be addressed by future loss- and gain-of-function and omics-level studies. While these data strongly implicate PI3K/AKT suppression as a primary mechanism, comprehensive validation via direct kinase inhibition assays and multiomics approaches (proteomics, phosphoproteomics, and transcriptomics) is warranted. Such studies would further elucidate the broader biological consequences of this signaling blockade and fully characterize the downstream effectors of the AKT signaling switch-off in both in vitro and in vivo systems.

The combination of 2,3′-dihydroxy-5′-methoxystilbene and cisplatin modulates NSCLC cell sensitivity in a context-dependent manner, emphasizing the dual potential and complexity of natural compounds as chemosensitizers. In A549 cells, the observed synergy (CI = 0.83) suggested the disruption of intrinsic resistance mechanisms. Previous studies have indicated that A549 cells acquire cisplatin resistance through enhanced DNA repair capacity and attenuated G2/M arrest, processes mediated by the ATM/p53/p21 axis [43]. The synergistic cytotoxicity observed in this study implies that the stilbene derivative may potentiate the efficacy of cisplatin by impairing DNA damage responses or facilitating a pro-apoptotic threshold. This aligns with the findings for resveratrol, which sensitizes A549 cells by promoting ROS generation and oxidative stress, thereby overcoming drug resistance [44]. In H460 cells, the interaction was additive to mildly synergistic (CI = 0.94). Given that H460 cells may exhibit a resistance phenotype driven by constitutive PI3K/AKT signaling, the ability of stilbene to inhibit this pathway (as demonstrated in Section 2.8) likely counteracts survival signaling, preventing the cells from neutralizing the chemotherapeutic insult. In contrast to A549 and H460 cells, H23 cells exhibited clear antagonism (CI = 1.32), indicating that this combination can be detrimental in certain molecular contexts. One plausible explanation for this phenomenon is that Nrf2/HO-1-mediated cisplatin and stilbenes are both capable of generating ROS; in some settings, this contributes to chemosensitization, but excessive ROS can strongly activate Nrf2/HO-1 and other antioxidant or detoxification programs that promote chemoresistance. In H23 cells, where 2,3′-dihydroxy-5′-methoxystilbene alone induced robust ROS accumulation, co-treatment with cisplatin elevated oxidative stress, driving the upregulation of Nrf2/HO-1, glutathione synthesis, detoxifying enzymes, and possibly Nrf2-dependent autophagy, thereby enhancing cisplatin tolerance and resulting in an apparent antagonistic interaction between the two agents. This is consistent with reports that Nrf2 activation can confer cisplatin resistance via cytoprotective pathways [45]. Although specific data on stilbene antagonism in H23 cells are limited, this poor response may reflect heightened drug efflux transporter activity, competing apoptotic pathways, and distinct redox buffering capacities. Collectively, these divergent interaction profiles highlight the critical necessity for molecular stratification; identifying predictive biomarkers is essential to select patient subsets that will benefit from such combinatorial regimens while avoiding antagonistic outcomes.

In conclusion, this study is the first to elucidate the selective anticancer activity of 2,3′-dihydroxy-5′-methoxystilbene in non-small-cell lung cancer (NSCLC). We demonstrated that this mono-methoxylated trans-stilbene exerts multifaceted inhibitory effects on proliferation, clonogenicity, migration, and survival. These effects are mediated by the modulation of the PI3K/AKT/GSK3β signaling axis and the disruption of redox homeostasis, as evidenced by the decreased phosphorylation of AKT and GSK3β. The precise causal contribution of this pathway to the observed antiproliferative and anti-invasive effects remains to be confirmed in future loss-of-function studies. Notably, the therapeutic efficacy was contingent on the specific cellular antioxidant context. H23 and H460 cells, characterized by compromised Nrf2/HO-1 defense mechanisms, exhibited pronounced sensitivity, whereas A549 cells displayed relative resistance owing to robust redox buffering. These findings provide a structural and mechanistic rationale for developing stilbene-based therapeutics tailored to tumors with AKT gain-of-function mutations and specific redox vulnerabilities. Future studies integrating phosphoproteomics, transcriptomics, and genetic manipulation of key network hubs (e.g., PIK3CA, SRC, and HSP90AA1) are required to delineate the precise signaling circuitry through which 2,3′-dihydroxy-5′-methoxystilbene exerts its AKT-mediated antitumor effects. To advance the translational potential of this lead compound, future investigations must extend beyond in vitro models to include in vivo efficacy validation, pharmacokinetic profiling, and scaffold optimization strategies aimed at overcoming resistance in refractory phenotypes such as A549. Moreover, a preliminary in vitro selectivity toward NSCLC cells over NIH/3T3 fibroblasts was observed, which warrants confirmation in normal human lung epithelial models.

## 4. Materials and Methods

### 4.1. 2,3′-Dihydroxy-5′-Methoxystilbene Preparation

2,3′-Dihydroxy-5′-methoxystilbene (Figure 1) was isolated from the roots and leaves of P. dianthum, as previously described [46]. For 2,3′-dihydroxy-5′-methoxystilbene preparation, it was dissolved in DMSO as a stock solution, which was further diluted with the cell culture medium to the desired working concentrations, and the control samples were incubated with 0.1% DMSO in the culture medium. The final DMSO concentration was less than 0.1%, indicating no toxicity. Control cells exposed to equal concentrations of DMSO were used for comparison with the 2,3′-dihydroxy-5′-methoxystilbene-treated group.

### 4.2. Cell Culture

Human non-small-cell lung cancer cell lines H460 (HTB-177), H23 (CRL-5800), and A549 (CCL-185), and the normal mouse embryonic fibroblast cell line NIH/3T3 (CRL-1658) were obtained from the American Culture Collection (ATCC, Manassas, VA, USA). H460 and H23 cells were cultured in RPMI-1640 medium, whereas A549 cells were maintained in DMEM (Gibco, Gaithersburg, MA, USA). All media contained 10% fetal bovine serum, 2 mM L-glutamine, and 100 U/mL of penicillin–streptomycin. Cultures were incubated at 37 °C with 5% CO_2_ until reaching 70–80% confluence prior to experiments.

### 4.3. Cytotoxicity and Cell Proliferation Assays

The cytotoxic effects of 2,3′-dihydroxy-5′-methoxystilbene were evaluated using the MTT colorimetric assay, according to previously established methods [18,47]. Cells were seeded in 96-well plates at a density of 1 × 10^4^ cells/well and incubated overnight at 37 °C in a humidified atmosphere with 5% CO_2_. The following day, the cells were treated with serial dilutions of 2,3′-dihydroxy-5′-methoxystilbene (0–200 μM) or cisplatin (0–250 μM) as a positive control for 24 and 48 h. Following treatment, the cells were washed twice with phosphate-buffered saline (PBS), and 100 μL of MTT solution (0.5 mg/mL in PBS) was added to each well. The plates were incubated for 3 h at 37 °C to allow formazan crystal formation. The medium was then removed, and 200 μL of dimethyl sulfoxide (DMSO) was added to each well to dissolve the formazan crystals. Absorbance was measured at 570 nm using a microplate reader (Perkin Elmer VICTOR3/Wallac1420).

For cell proliferation assays, cells were seeded at a density of 2 × 10^3^ cells/well in 96-well plates and allowed to adhere overnight. Cells were subsequently treated with 2,3′-dihydroxy-5′-methoxystilbene for 48 h, which corresponds to approximately two cell division cycles. Cell proliferation was assessed using the MTT assay, as described above. The results are expressed as the percentage of cell proliferation in the treated wells compared to that in the control.

### 4.4. Cell Migration Assays

Cell migration was assessed using a wound healing assay, as previously described, with minor modifications [18,48]. This assay was performed to evaluate the inhibitory effects of 2,3′-dihydroxy-5′-methoxystilbene on the migratory capacity of NSCLC cell lines H23, H460, and A549. Cells were seeded in 24-well plates at a density of 2 × 10^5^ cells per well and cultured overnight in complete medium until confluence was achieved. A standardized linear scratch was created across the cell monolayer using a sterile 200 μL pipette tip to simulate a wound. Detached cells and cellular debris were removed by gentle washing with phosphate-buffered saline (PBS) (500 μL per well). Subsequently, the cells were treated with various concentrations of 2,3′-dihydroxy-5′-methoxystilbene (0–40 μM) and incubated under standard culture conditions. Wound closure was monitored and documented using phase-contrast microscopy with a Nikon Ts2 inverted microscope (Nikon, Tokyo, Japan). Digital images were captured at 0, 24, and 48 h after scratch induction. The wound area was quantified using ImageJ software version 8 (National Institutes of Health, Bethesda, MD, USA), and the percentage of wound closure was calculated. The migration rate was determined from the slope of the linear regression analysis of wound closure over a 48 h. observation period. All experiments were performed in triplicate to ensure reproducibility.

### 4.5. Anchorage-Independent Growth Assay

The inhibitory effect of 2,3′-dihydroxy-5′-methoxystilbene on anchorage-independent growth was evaluated using a soft agar colony formation assay, as previously described with minor modifications [49,50]. This assay was performed using the NSCLC cell lines A549, H23, and H460. Briefly, 1.2% and 0.6% noble agar was melted and kept at 55 °C in a water bath. A two-layer soft agar system was prepared in 24-well plates (Corning Inc., Corning, NY, USA). The bottom layer was a mixture of 1.2% agarose and complete DMEM or RPMI medium supplemented with 5% FBS at a 1:1 ratio, respectively. After adding 250 μL/well of the mixture into a 24-well plate, the agar was solidified at 37 °C for 2 h. The upper layer was prepared from 0.6% noble agar and a single cell containing 1.5 × 10^3^ cells/well (250 μL) suspension of A549, H23, and H460 cells to give a final concentration of 0.33% agarose. The layer was allowed to solidify at 37 °C for 1 h. Subsequently, either non-cytotoxic concentrations of 2,3′-dihydroxy-5′-methoxystilbene or 0.1% (*v*/*v*) DMSO was added to the cultures, which were incubated at 37 °C in a humidified atmosphere with 5% CO_2_ for 14 days. The culture medium was refreshed every three days throughout the incubation period. At the end of the incubation period, colonies were visualized using an inverted light microscope equipped with a digital camera system. Digital images were captured and analyzed to determine colony numbers with a diameter ≥50 μm using ImageJ software version 8, and the mean colony number per well is expressed as a percentage of the control. For colony size analysis, images were thresholded, and individual colony areas were quantified using ImageJ software version 8. The mean colony area and size distribution were calculated for each treatment group. Quantitative analysis was performed using AxioVision 4 software (Carl Zeiss MicroImaging GmbH, Jena, Germany) and ImageJ software version 8 (National Institutes of Health, Bethesda, MD, USA).

### 4.6. Determination of Reactive Oxygen Species (ROS)

Intracellular reactive oxygen species levels were measured using the 2′,7′-dichlorodihydrofluorescein diacetate (DCFH-DA) fluorometric assay [47]. Cells were seeded at a density of 7.5 × 10^4^ cells/well in 24-well plates and incubated overnight under standard culture conditions. After two washes with PBS, the cells were loaded with 100 μM DCFH-DA solution and incubated for 30 min at 37 °C in the dark. Subsequently, cells were treated with different concentrations of 2,3′-dihydroxy-5′-methoxystilbene (0–40 μM) or 600 μM hydrogen peroxide (H_2_O_2_) as a positive control for 1 h. After treatment, the cells were lysed using 1% (*v*/*v*) Triton X-100 lysis buffer. The fluorescence intensity of oxidized 2′,7′-dichlorofluorescein (DCF) was measured using a microplate reader (Perkin Elmer VICTOR3/Wallac1420) at excitation and emission wavelengths of 485 and 535 nm, respectively. The results are expressed as fold changes relative to those of the untreated control cells.

### 4.7. Cell Apoptosis Analysis

Apoptotic cell death was assessed using Hoechst 33342 staining, according to the established protocols [18,51,52]. A549, H23, and H460 cells were seeded at a density of 8 × 10^3^ cells per well in 96-well plates and allowed to adhere overnight. After treatment with various concentrations of 2,3′-dihydroxy-5′-methoxystilbene for 48 h, the cells were washed with PBS and incubated with Hoechst 33342 (10 μM) for 30 min at room temperature in the dark. Apoptotic nuclear morphological changes, including chromatin condensation and nuclear fragmentation, were visualized using a fluorescence microscope (BX-FLA; Olympus, Tokyo, Japan) at 20× magnification, with excitation and emission wavelengths of 350 and 461 nm, respectively. The percentage of apoptotic cells was determined by counting the cells exhibiting characteristic apoptotic nuclear morphology relative to the total cell population in at least five randomly selected fields per well. All experiments were performed in triplicates.

### 4.8. Western Blot Analysis

For protein expression analysis, 8 × 10^5^ cells were seeded in 6-well plates and incubated overnight to allow cell attachment. The cells were then treated with various concentrations of 2,3′-dihydroxy-5′-methoxystilbene for 48 h. Following treatment, the cells were harvested and lysed on ice for 30 min using radioimmunoprecipitation assay (RIPA) buffer supplemented with a protease inhibitor cocktail (Corning Inc., Corning, NY, USA). Cell lysates were clarified by centrifugation at 12,000× g for 15 min at 4 °C, and the resulting supernatants were collected. Total protein concentration was determined using a bicinchoninic acid (BCA) protein assay kit (Thermo Fisher Scientific, Waltham, MA, USA). Protein samples were denatured by boiling in 2× Laemmli sample buffer at 95 °C for 5 min. Equal amounts of total protein (40 μg per lane) were resolved using SDS-PAGE and transferred onto polyvinylidene difluoride (PVDF) membranes (Millipore, Burlington, MA, USA). Membranes were blocked with 5% (*w*/*v*) non-fat dry milk in Tris-buffered saline containing 0.1% Tween-20 (TBS-T) for 1 h at room temperature. After blocking, the membranes were incubated overnight at 4 °C with primary rabbit antibodies against phospho-Akt (Ser473, 1:1000), Akt (1:1000), phospho-GSK-3β (Ser9, 1:1000), and GSK-3β (1:1000) (all from CST, Beverly, MA, USA), diluted in blocking buffer. Following washing with TBS-T, the membranes were incubated with horseradish peroxidase (HRP)-conjugated secondary antibodies for 2 h at room temperature. Glyceraldehyde-3-phosphate dehydrogenase (GAPDH) served as the internal loading control. Protein bands were detected using enhanced chemiluminescence (ECL) with Immobilon Western Chemiluminescent HRP Substrate (Millipore, Burlington, MA, USA) and visualized using a ChemiDoc Imaging System. Immunoblot intensities were quantified using ImageJ software version 8 (National Institutes of Health, Bethesda, MD, USA) and normalized to the levels of GAPDH.

### 4.9. Gene Ontology (GO) and Kyoto Encyclopedia of Genes and Genomes (KEGG) Pathway Analysis

Gene Ontology (GO) functional enrichment analysis [53] and Kyoto Encyclopedia of Genes and Genomes (KEGG) pathway analysis [54,55] were performed using the interactive tool ShinyGO version 0.85.1, which has been updated to Ensembl Release 113, and STRING-db v12 [56]. Dot plots were used to visualize the significantly enriched GO terms and KEGG pathways based on gene count and -log10 FDR. Potential molecular targets of 2,3′-dihydroxy-5′-methoxystilbene were predicted using molecular docking analysis of proteins involved in the phosphatidylinositol 3-kinase (PI3K)/protein kinase B (Akt) signaling pathway. These predictions were validated using structural information from the PubChem database and the biological activity profiles from the ChEMBL database. Functional annotation and statistical analyses were performed using standard bioinformatics tools, with a significance threshold of *p* < 0.05. These integrative analyses provided mechanistic insights into the multi-target effects of 2,3′-dihydroxy-5′-methoxystilbene, highlighting its regulatory role in key biological processes, such as apoptosis and cell proliferation, as well as critical signaling pathways, including PI3K/Akt, which are relevant to NSCLC pathogenesis.

### 4.10. Protein–Protein Interaction (PPI) Network Construction and Molecular Docking Analysis with AKT

To investigate the protein interaction landscape associated with 2,3′-dihydroxy-5′-methoxystilbene treatment, a protein–protein interaction (PPI) network was constructed using the STRING database (version 11.0) [57,58]. Gene targets of interest were analyzed using the Homo sapiens organism model with a high confidence interaction score threshold of 0.950. In the resulting network visualization, nodes represent individual proteins, and edges indicate functional protein–protein associations [59]. Key target proteins were identified through comprehensive topological analysis, including calculations of degree centrality, betweenness centrality, closeness centrality, and clustering coefficient parameters using Cytoscape software (version 3.8).

For molecular docking studies, the three-dimensional X-ray crystallographic structure of activated protein kinase B (AKT) was retrieved from the Protein Data Bank (PDB ID: 1O6L). The chemical structure of 2,3′-dihydroxy-5′-methoxystilbene was generated using ChemDraw Ultra 17.0 software (PerkinElmer, Waltham, MA, USA), and the canonical simplified molecular input line entry system (SMILES) notation was obtained from the Chemical Entities of Biological Interest (ChEBI) database [60]. The binding interactions between 2,3′-dihydroxy-5′-methoxystilbene and AKT protein were evaluated using computational molecular docking simulations. Potential binding sites on the AKT protein surface were identified using the CurPocket algorithm, which employs curvature-based cavity detection methods. Molecular docking calculations were performed using AutoDock Vina (version 1.1.2) integrated within the CB-Dock2 platform, which enables automated blind docking simulations [59]. The binding pose exhibiting the most favorable (most negative) Vina score, which is indicative of the highest predicted binding affinity, was selected for detailed structural analysis. Comprehensive binding parameters, including docking center coordinates, grid box dimensions, interacting amino acid residues, and intermolecular contacts, were thoroughly examined. The resulting protein–ligand binding interactions and three-dimensional docking conformations were visualized and analyzed using BIOVIA Discovery Studio Visualizer software (BIOVIA, San Diego, CA, USA), providing detailed structural insights into the molecular binding mechanism of 2,3′-dihydroxy-5′-methoxystilbene with AKT protein.

### 4.11. Calculation of the Combination Index

To determine the combined effects of 2,3′-dihydroxy-5′-methoxystilbene and cisplatin on non-small-cell lung cancer cells, non-small-cell lung cancer (NSCLC) cell lines A549, H23, and H460 were exposed to increasing concentrations of cisplatin (0–50μM), either as a single agent or in combination with a fixed concentration (20 μM) of 2,3′-dihydroxy-5′-methoxystilbene. Both compounds were added simultaneously, and the cells were incubated for 48 h. Cell viability was measured using the MTT assay. Dose–response curves for each drug, alone and in combination, were constructed using GraphPad Prism version 8. Combination effects were analyzed according to the Chou–Talalay method, and the median-effect equation was used to characterize dose–effect relationships and calculate the combination index (CI) for each drug pair at a fixed effect level [61,62]. The IC_50_ values for cisplatin and stilbene, alone and in combination, were plotted to facilitate a visual comparison with the theoretical additive line. CI values were manually computed for each combination point to classify the drug interactions as synergistic (CI < 1), additive (CI = 1), or antagonistic (CI > 1). All experiments were performed in triplicate to ensure reproducibility.

### 4.12. Statistical Analysis

All experimental data are presented as mean ± standard error of the mean (SEM) values derived from a minimum of four independent biological replicates. Statistical analyses were performed using GraphPad Prism version 8 (GraphPad Software Inc., San Diego, CA, USA). Statistical significance between experimental groups was determined using one-way analysis of variance (ANOVA), followed by Tukey’s multiple comparison post hoc test. Statistical significance was set at *p* < 0.05.

## 5. Conclusions

This study evaluated the anticancer activity of 2,3′-dihydroxy-5′-methoxystilbene, a naturally occurring stilbene derivative, in non-small-cell lung cancer (NSCLC) cell lines A549, H23, and H460. The compound exhibited selective cytotoxicity, demonstrating the strongest inhibitory effects on H23 and H460 cells, while showing no significant toxicity toward normal NIH/3T3 fibroblasts. It suppressed anchorage-independent growth and migration in a cell line-dependent manner, with H23 and H460 cells showing greater sensitivity than A549 cells did. The stilbene derivative induced oxidative stress and apoptosis, particularly in H23 cells, which have a potentially lower antioxidant capacity. Computational analyses predicted its selective binding to activated AKT and modulation of the PI3K/AKT/GSK3β pathway. This study highlights the potential of 2,3′-dihydroxy-5′-methoxystilbene as a targeted therapeutic candidate for NSCLC, particularly in the H23 and H460 subtypes, through the inhibition of proliferation, clonogenicity, and migration, and induction of apoptosis via the PI3K/AKT signaling pathway.

## Figures and Tables

**Table 1:** IC_50_ values of 2,3′-dihydroxy-5′-methoxystilbene and cisplatin against NSCLC cell lines and normal fibroblasts.

NSCLC Cell Lines/Normal Mouse Fibroblast Embryonic Cell Lines	^a^ IC_50_ (μM ± SEM)			
	2,3′-Dihydroxy-5′-Methoxystilbene		Cisplatin	
	24 h	48 h	24 h	48 h
A549	57.68 ± 4.93	37.03 ± 5.96	49.57 ± 5.26	17.44 ± 1.33
H23	44.79 ± 5.92	23.39 ± 3.27	97.87 ± 8.99	28.26 ± 2.19
H460	51.14 ± 3.91	24.20 ± 2.61	70.46 ± 6.36	30.12 ± 3.27
NIH/3T3	>100	>100	38.62 ± 4.46	16.49 ± 1.97

^a^ Concentration that inhibits cell viability by 50%. Data are expressed as the mean ± standard error of the mean (SEM) (*n* = 3).

## Data Availability

The datasets generated during and/or analyzed during the current study are available by request; please contact the corresponding authors.

## Supplementary Materials

The following supporting information can be downloaded at https://www.mdpi.com/article/10.3390/ijms27020719/s1.

## Abbreviations

The following abbreviations are used in this manuscript:

AKT	Activated protein kinase B
GSK3β	Glycogen synthase kinase 3 beta
NSCLC	Non-small-cell lung cancer
IC_50_	Half-maximal inhibitory concentration
CI	Combination index
GO	Gene Ontology
KEGG	Kyoto Encyclopedia of Genes and Genomes
PPI	Protein–protein interaction
PDB	Protein Data Bank
DCFH-DA	2′,7′-dichlorodihydrofluorescein diacetate
DCF	2′,7′-dichlorofluorescein
MTT	3-(4,5-dimethylthiazol-2-yl)-2,5-diphenyltetrazolium bromide
GAPDH	Glyceraldehyde-3-phosphate dehydrogenase
SDS-PAGE	Sodium dodecyl sulfate polyacrylamide gel electrophoresis
PVDF	Polyvinylidene difluoride
ANOVA	One-way analysis of variance
SEM	Standard error of the mean
Nrf2	Nuclear factor erythroid 2
HO-1	Heme oxygenase-1
ROS	Reactive oxygen species
