# Supplementary material for: Anticancer Activity of 2,3′-Dihydroxy-5′-Methoxystilbene Against NSCLC Cell Lines Through AKT-Dependent Mechanisms: A Comprehensive In Vitro and Computational Analysis
Source: Int J Mol Sci. 2026 Jan 10;27(2):719. doi: 10.3390/ijms27020719 (PMC12840911; doi:10.3390/ijms27020719)
Supplement: Supplementary file 1 [file ijms-27-00719-s001.zip › ijms-4034490-supplementary.pdf]

# Supplementary Materials

**Table S1** Target prediction of Dihydroxy methoxystillbene by Swisstargetprediction Tool

| | | | |
|---|---|---|---|
| Estrogen receptor alpha | ESR1 | Leukocyte elastase | ELANE |
| Cyclooxygenase-1 | PTGS1 | Glycogen synthase kinase-3 beta | GSK3B |
| Cyclooxygenase-2 | PTGS2 | Serine/threonine-protein kinase PLK1 | PLK1 |
| Quinone reductase 2 | NQO2 | Serine/threonine-protein kinase PLK4 | PLK4 |
| Aryl hydrocarbon receptor | AHR | Serine/threonine-protein kinase WEE1 | WEE1 |
| Nuclear factor NF-kappa-B p65 subunit | RELA | Cytochrome P450 11B1 | CYP11B1 |
| Cytochrome P450 19A1 | CYP19A1 | Cytochrome P450 11B2 | CYP11B2 |
| Beta amyloid A4 protein | APP | Dual specificty protein kinase CLK1 | CLK1 |
| Cytochrome P450 1B1 | CYP1B1 | Dual specificity tyrosine-phosphorylation-regulated kinase 1B | DYRK1B |
| Tubulin beta-1 chain | TUBB1 | Dynamin-1 | DNM1 |
| P-glycoprotein 1 | ABCB1 | Tyrosine-protein kinase LCK | LCK |
| Cytochrome P450 1A1 | CYP1A1 | PI3-kinase p110-beta subunit | PIK3CB |
| Cytochrome P450 1A2 | CYP1A2 | Insulin-like growth factor I receptor | IGF1R |
| Tubulin beta-3 chain | TUBB3 | Serine/threonine-protein kinase PIM1 | PIM1 |
| Microtubule-associated protein tau | MAPT | Tyrosine-protein kinase SRC | SRC |
| Carbonic anhydrase II | CA2 | Serine/threonine-protein kinase PIM2 | PIM2 |
| Carbonic anhydrase VII | CA7 | Tyrosinase | TYR |
| Carbonic anhydrase I | CA1 | Dual specificity mitogen-activated protein kinase kinase 1 | MAP2K1 |

| | | | |
|---|---|---|---|
| Carbonic anhydrase III | CA3 | Testis-specific androgen-binding protein | SHBG |
| Carbonic anhydrase VI | CA6 | Heat shock protein HSP 90-alpha | HSP90AA1 |
| Carbonic anhydrase XII | CA12 | Dual specificity phosphatase Cdc25B | CDC25B |
| Carbonic anhydrase XIV | CA14 | Nitric-oxide synthase, brain | NOS1 |
| Carbonic anhydrase IX | CA9 | Nitric-oxide synthase, endothelial | NOS3 |
| Carbonic anhydrase IV | CA4 | Cytochrome P450 2C19 | CYP2C19 |
| Carbonic anhydrase XIII | CA13 | Glucagon receptor | GCGR |
| Carbonic anhydrase VB | CA5B | Kinesin-1 heavy chain/ Tyrosine-protein kinase receptor RET | RET |
| Carbonic anhydrase VA | CA5A | Cytochrome P450 17A1 | CYP17A1 |
| Monoamine oxidase A | MAOA | 3-phosphoinositide dependent protein kinase-1 | PDPK1 |
| HMG-CoA reductase | HMGCR | Metabotropic glutamate receptor 5 (by homology) | GRM5 |
| Cyclin-dependent kinase 5/CDK5 activator 1 | CDK5R1 CDK5 | Glutathione S-transferase A1 | GSTA1 |
| Dual-specificity tyrosine-phosphorylation regulated kinase 1A (by homology) | DYRK1A | Heat shock protein HSP 90-beta | HSP90AB1 |
| Epidermal growth factor receptor erbB1 | EGFR | Serine/threonine-protein kinase Chk1 | CHEK1 |
| Arachidonate 5-lipoxygenase | ALOX5 | Uridine phosphorylase 1 (by homology) | UPP1 |
| PI3-kinase p110-alpha subunit | PIK3CA | LDL-associated phospholipase A2 | PLA2G7 |
| Norepinephrine transporter | SLC6A2 | Cyclin-dependent kinase 1/cyclin B | CCNB3 CDK1 CCNB1 CCNB2 |

| | | | |
|---|---|---|---|
| Serine/threonine-protein kinase Nek1 | NEK1 | Urokinase-type plasminogen activator | PLAU |
| Alkaline phosphatase, tissue-nonspecific isozyme | ALPL | Poly [ADP-ribose] polymerase-1 | PARP1 |
| Serine/threonine-protein kinase RAF | RAF1 | Protein kinase C (PKC) | PRKCZ |
| Ribosomal protein S6 kinase alpha 3 | RPS6KA3 | Dual specificity phosphatase Cdc25A | CDC25A |
| Serine/threonine-protein kinase B-raf | BRAF | Neprilysin (by homology) | MME |
| Tyrosine-protein kinase SYK | SYK | NADPH oxidase 4 | NOX4 |
| Dual specificity protein phosphatase 3 | DUSP3 | Tyrosine-protein kinase receptor FLT3 | FLT3 |
| Phospholipase A-2-activating protein | PLAA | Steryl-sulfatase | STS |
| PI3-kinase p110-gamma subunit | PIK3CG | Ephrin receptor | EPHB4 |
| Serine/threonine-protein kinase Aurora-B | AURKB | Catechol O-methyltransferase | COMT |
| Cyclin-dependent kinase 2 | CDK2 | Presequence protease, mitochondrial | PITRM1 |
| Cyclin-dependent kinase 1 | CDK1 | Cytochrome P450 3A4 | CYP3A4 |
| Cyclin-dependent kinase 4 | CDK4 | Serine/threonine-protein kinase ILK-1 | ILK |
| Serine/threonine-protein kinase Aurora-A | AURKA | Lysine-specific histone demethylase 1 | KDM1A |
| Plasminogen activator inhibitor-1 | SERPINE1 | Sodium/hydrogen exchanger 1 | SLC9A1 |

**Table S2** Target prediction of Dihydroxy methoxystillbene by Similarity ensemble approach (SEA) Tool

| | | | |
|---|---|---|---|
| Nuclear factor erythroid 2-related factor 2 | NFE2L2 | Xanthine dehydrogenase/oxidase | XDH |

| | | | |
|---|---|---|---|
| Cytochrome P450 1B1 | CYP1B1 | Amine oxidase [flavin-containing] B | MAOB |
| Cytochrome P450 1A1 | CYP1A1 | Aryl hydrocarbon receptor | AHR |
| Endothelin-converting enzyme 2 | ECE2 | Potassium voltage-gated channel subfamily D member 3 | KCND3 |
| Tubulin beta-1 chain | TUBB1 | Prostaglandin G/H synthase 1 | PTGS1 |
| 25-hydroxyvitamin D-1 alpha hydroxylase, mitochondrial | Cyp27b1 | Kinesin-like protein KIF20A | KIF20A |
| 1,25-dihydroxyvitamin D(3) 24-hydroxylase, mitochondrial | CYP24A1 | Cystathionine beta-synthase | CBS |
| Sterol 26-hydroxylase, mitochondrial | CYP27A1 | Calmodulin-1 | CALM1 |
| Amyloid-beta precursor protein | APP | Arachidonate 5-lipoxygenase | ALOX5 |
| Broad substrate specificity ATP-binding cassette transporter ABCG2 | ABCG2 | Prostaglandin G/H synthase 2 | PTGS2 |
| Transthyretin | TTR | PH domain leucine-rich repeat-containing protein phosphatase 2 | PHLPP2 |
| Transcription factor p65 | RELA | Tumor necrosis factor receptor superfamily member 1A | TNFRSF1 A |
| Microtubule-associated protein tau | MAPT | Amine oxidase [flavin-containing] A | MAOA |
| Nuclear factor NF-kappa-B p105 subunit | NFKB1 | Glucose-6-phosphatase | G6PC |
| Glucose-6-phosphate exchanger SLC37A4 | SLC37A4 | Lactoylglutathione lyase | GLO1 |
| Ribosyldihydronicotinamid e dehydrogenase [quinone] | NQO2 | Potassium channel subfamily K member 9 | KCNK9 |
| Ornithine decarboxylase | CXCL12 | Proto-oncogene c-Fos | FOS |
| Carbonyl reductase [NADPH] 1 | ODC1 | Sentrin-specific protease 7 | SENP7 |

| | | | |
|---|---|---|---|
| CDGSH iron-sulfur domain-containing protein 1 | CISD1 | Transcription factor AP-1 | JUN |
| Serine/threonine-protein kinase 17B | STK17B | Serine/threonine-protein kinase/endoribonuclease IRE1 | ERN1 |
| Aldo-keto reductase family 1 member B10 | AKR1B10 | Tyrosine-protein phosphatase non-receptor type 6 | PTPN6 |
| Prostaglandin E synthase 2 | PTGES2 | Macrophage migration inhibitory factor | MIF |
| NF-kappa-B essential modulator | IKBKG | Transcription factor AP-1 | JUN |

**Table S3** Pharmacokinetic prediction by pkCSM Tool

| **Property** | **Model Name** | **Predicted Value** | **Unit** |
|---|---|---|---|
| Absorption | Water solubility | -3.441 | Numeric (log mol/L) |
| | Caco2 permeability | 1.267 | Numeric (log Papp in $10^{-6}$ cm/s) |
| | Intestinal absorption | 91.123 | Numeric (% Absorbed) |
| | Skin Permeability | -2.768 | Numeric (log Kp) |
| | P-glycoprotein substrate | Yes | Categorical (Yes/No) |
| | P-glycoprotein I inhibitor | No | Categorical (Yes/No) |
| | P-glycoprotein II inhibitor | No | Categorical (Yes/No) |
| Distribution | VDss | 0.171 | Numeric (log L/kg) |
| | Fraction unbound | 0.05 | Numeric (Fu) |
| | BBB permeability | 0.097 | Numeric (log BB) |
| | CNS permeability | -2.029 | Numeric (log PS) |
| Metabolism | CYP2D6 substrate | No | Categorical (Yes/No) |
| | CYP3A4 substrate | Yes | Categorical (Yes/No) |
| | CYP1A2 inhibitior | Yes | Categorical (Yes/No) |
| | CYP2C19 inhibitior | Yes | Categorical (Yes/No) |
| | CYP2C9 inhibitior | No | Categorical (Yes/No) |
| | CYP2D6 inhibitior | No | Categorical (Yes/No) |
| | CYP3A4 inhibitior | No | Categorical (Yes/No) |

| | | | |
|---|---|---|---|
| Excretion | Total Clearance | 0.257 | Numeric (log ml/min/kg) |
| | Renal OCT2 substrate | No | Categorical (Yes/No) |
| Toxicity | AMES toxicity | Yes | Categorical (Yes/No) |
| | Max. tolerated dose | -0.069 | Numeric (log mg/kg/day) |
| | hERG I inhibitor | No | Categorical (Yes/No) |
| | hERG II inhibitor | No | Categorical (Yes/No) |
| | Oral Rat Acute Toxicity (LD50) | 2.236 | Numeric (mol/kg) |
| | Oral Rat Chronic Toxicity (LOAEL) | 1.63 | Numeric (log mg/kg_bw/day) |
| | Hepatotoxicity | No | Categorical (Yes/No) |
| | Skin Sensitisation | No | Categorical (Yes/No) |
| | *T.Pyriformis* toxicity | 1.041 | Numeric (log ug/L) |
| | Minnow toxicity | 0.793 | Numeric (log mM) |

**Table S4** List of intercept targets between compound and non-small cell lung cancer cell targets by Venny

| | | | |
|---|---|---|---|
| EGFR | FLT3 | CDC25B | HMGCR |
| PIK3CA | PLAU | MME | SHBG |
| BRAF | SERPINE1 | PDPK1 | NOS1 |
| CDK4 | CHEK1 | CYP1A2 | XDH |
| NFE2L2 | PLK1 | WEE1 | CA2 |
| MAP2K1 | FOS | AHR | SLC9A1 |
| RET | GSK3B | ODC1 | ERN1 |
| RAF1 | CYP19A1 | EPHB4 | PLK4 |
| PIK3CG | CYP1B1 | TUBB3 | GLO1 |
| ESR1 | CYP17A1 | CYP24A1 | DYRK1A |
| JUN | MIF | MAPT | GSTA1 |
| SRC | CDC25A | PTGS1 | MAOA |
| ABCB1 | TNFRSF1A | CYP2C19 | CALM1 |
| CDK2 | AURKB | KDM1A | RPS6KA3 |
| PTGS2 | PIK3CB | PTPN6 | PLA2G7 |
| NFKB1 | CA9 | PIM1 | CA12 |
| IGF1R | SYK | AKR1B10 | CA4 |
| AURKA | NOS3 | APP | PHLPP2 |
| CYP1A1 | ELANE | NOX4 | TUBB1 |
| CXCL12 | ALOX5 | TTR | GCGR |

| | | | |
|---|---|---|---|
| PARP1 | IKBKG | PRKCZ | DUSP3 |
| ABCG2 | CYP3A4 | CBR1 | DNM1 |
| CDK1 | LCK | CBS | CLK1 |
| HSP90AA1 | COMT | HSP90AB1 | CA1 |
| RELA | ILK | DYRK1B | ECE2 |
| CA14 | | | |

**Figure S1** Intercept targets between compound's targets and non-small cell lung cancer's targets by Venny

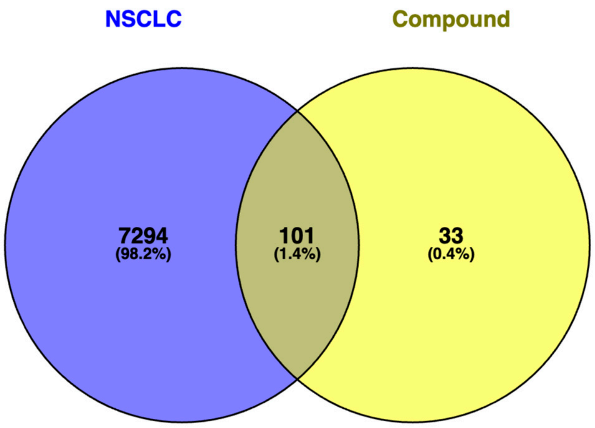

**Figure S2** Gene Ontology (GO) enrichment analysis based on biological process, molecular function, and cellular component. The data are ranged from the highest significant on the top to those of lower at the bottom.

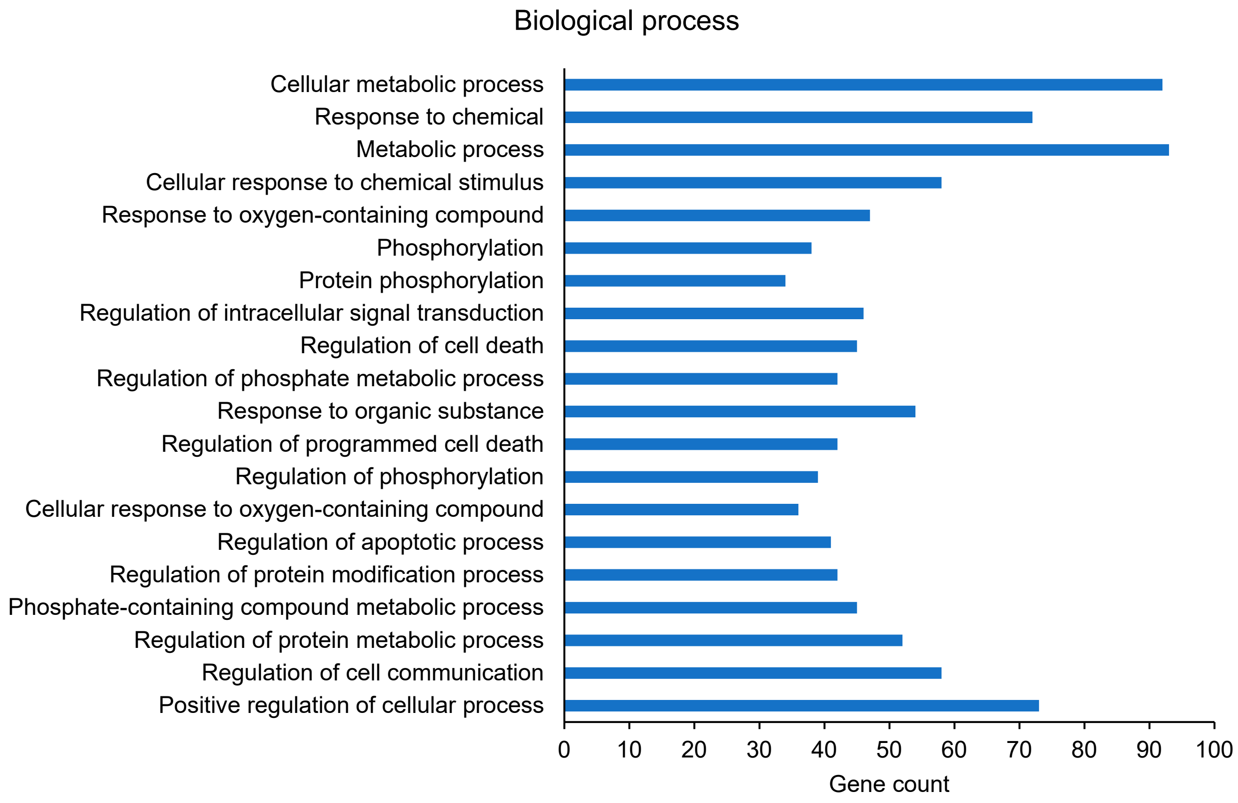

Biological process
Cellular metabolic process
Response to chemical
Metabolic process
Cellular response to chemical stimulus
Response to oxygen-containing compound
Phosphorylation
Protein phosphorylation
Regulation of intracellular signal transduction
Regulation of cell death
Regulation of phosphate metabolic process
Response to organic substance
Regulation of programmed cell death
Regulation of phosphorylation
Cellular response to oxygen-containing compound
Regulation of apoptotic process
Regulation of protein modification process
Phosphate-containing compound metabolic process
Regulation of protein metabolic process
Regulation of cell communication
Positive regulation of cellular process
0
10
20
30
40
50
60
70
80
90
100
Gene count

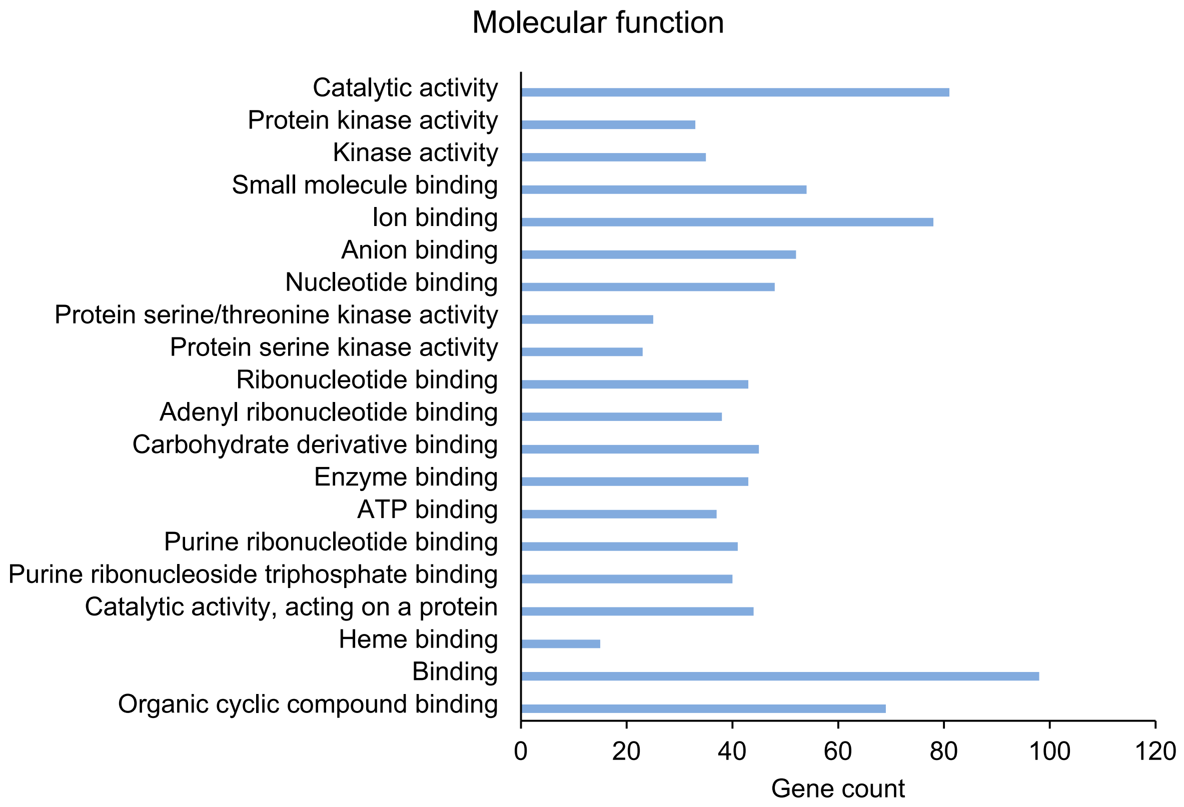
Molecular function
Catalytic activity
Protein kinase activity
Kinase activity
Small molecule binding
Ion binding
Anion binding
Nucleotide binding
Protein serine/threonine kinase activity
Protein serine kinase activity
Ribonucleotide binding
Adenyl ribonucleotide binding
Carbohydrate derivative binding
Enzyme binding
ATP binding
Purine ribonucleotide binding
Purine ribonucleoside triphosphate binding
Catalytic activity, acting on a protein
Heme binding
Binding
Organic cyclic compound binding
0
20
40
60
80
100
120
Gene count

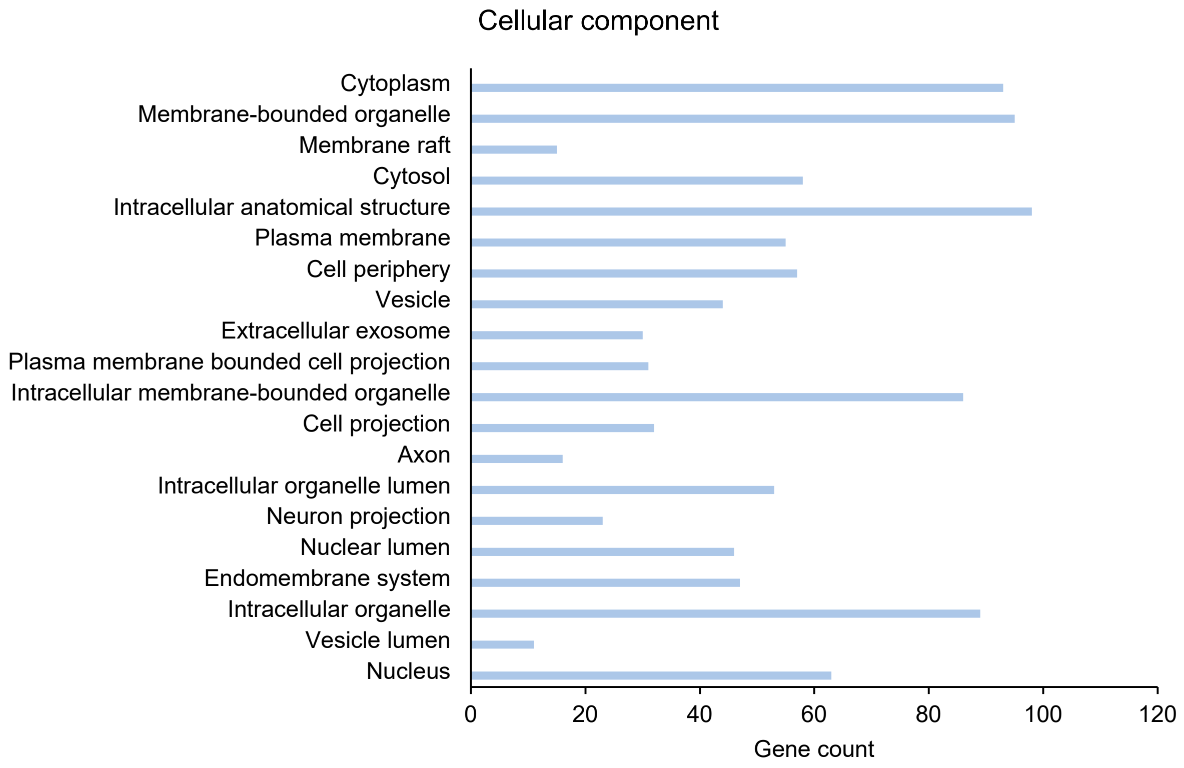
Cellular component
Cytoplasm
Membrane-bounded organelle
Membrane raft
Cytosol
Intracellular anatomical structure
Plasma membrane
Cell periphery
Vesicle
Extracellular exosome
Plasma membrane bounded cell projection
Intracellular membrane-bounded organelle
Cell projection
Axon
Intracellular organelle lumen
Neuron projection
Nuclear lumen
Endomembrane system
Intracellular organelle
Vesicle lumen
Nucleus
0
20
40
60
80
100
120
Gene count

**Figure S3** KEGG pathway analysis. The data are ranged from the highest significant on the top to those of lower at the bottom.

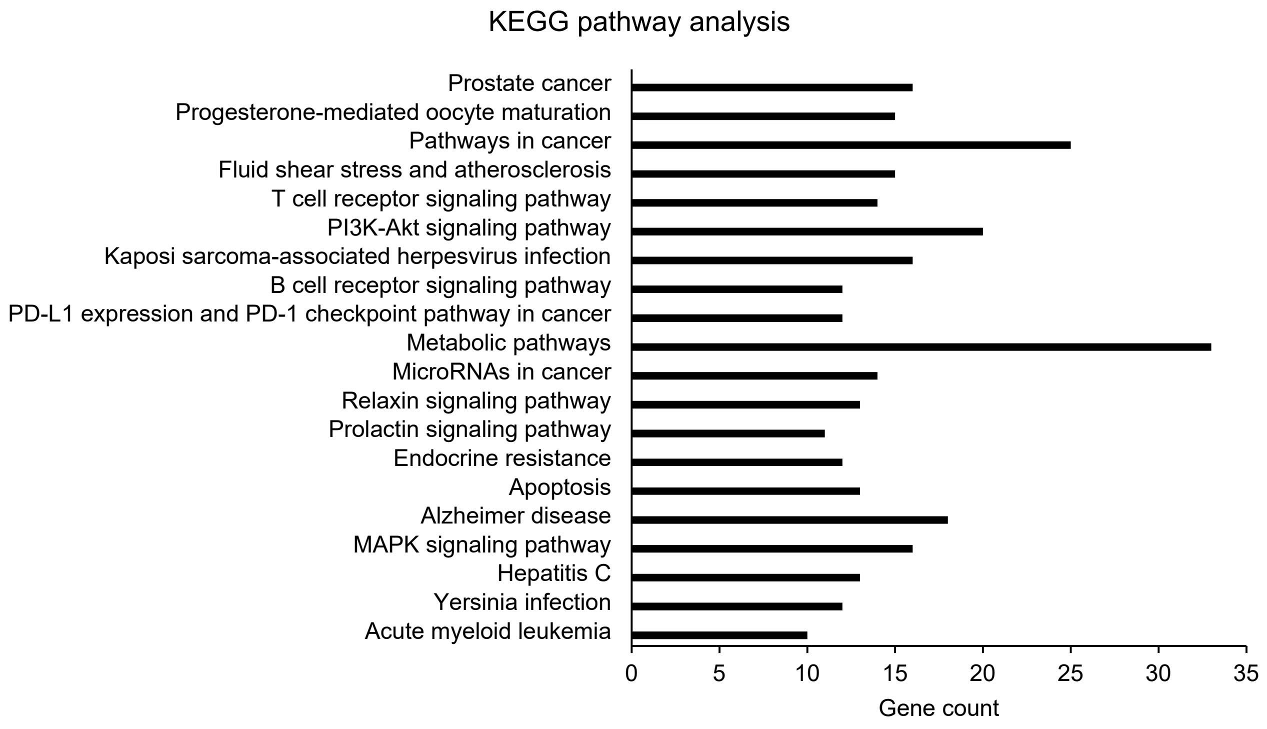

**Table S5** Important nodes in network analyzer

| Name | Degree | Betweenness Centrality | Closeness Centrality | Clustering Coefficient |
|---|---|---|---|---|
| ESR1 | 14 | 0.308224943 | 0.439189189 | 0.230769231 |
| HSP90AA1 | 14 | 0.350957212 | 0.442176871 | 0.230769231 |
| PIK3CA | 14 | 0.103734167 | 0.355191257 | 0.296703297 |
| SRC | 14 | 0.113670771 | 0.396341463 | 0.32967033 |
| HSP90AB1 | 12 | 0.121774519 | 0.419354839 | 0.303030303 |
| PIK3CB | 12 | 0.025568103 | 0.314009662 | 0.348484848 |
| CDK1 | 10 | 0.114086538 | 0.335051546 | 0.377777778 |
| RAF1 | 10 | 0.041404603 | 0.359116022 | 0.333333333 |
| CYP1A1 | 9 | 0.182700471 | 0.326633166 | 0.305555556 |
| GSK3B | 9 | 0.053464302 | 0.351351351 | 0.305555556 |
| EGFR | 9 | 0.036359215 | 0.380116959 | 0.527777778 |
| PLK1 | 8 | 0.075616987 | 0.331632653 | 0.392857143 |
| CYP3A4 | 8 | 0.121617619 | 0.294117647 | 0.357142857 |
| CDC25A | 7 | 0.002780449 | 0.26 | 0.619047619 |
| CYP1A2 | 7 | 0.040011828 | 0.268595041 | 0.380952381 |
| JUN | 7 | 0.019881865 | 0.347593583 | 0.380952381 |
| CYP2C19 | 6 | 0.09019765 | 0.238970588 | 0.333333333 |
| MAPT | 6 | 0.119711538 | 0.331632653 | 0.2 |
| BRAF | 6 | 0.012949233 | 0.347593583 | 0.6 |
| CDC25B | 6 | 3.61E-04 | 0.257936508 | 0.8 |
| WEE1 | 6 | 3.61E-04 | 0.257936508 | 0.8 |

| | | | | |
|---|---|---|---|---|
| PTPN6 | 6 | 0.01850353 | 0.299539171 | 0.666666667 |
| RELA | 6 | 0.004280504 | 0.328282828 | 0.466666667 |
| CDK2 | 5 | 0.001169872 | 0.257936508 | 0.7 |
| FOS | 5 | 9.51E-04 | 0.329949239 | 0.7 |
| IGF1R | 5 | 0.002240572 | 0.333333333 | 0.9 |
| NFKB1 | 5 | 0.003198773 | 0.326633166 | 0.5 |
| PRKCZ | 5 | 0.008488669 | 0.305164319 | 0.6 |
| IKBKG | 5 | 0.013082647 | 0.321782178 | 0.2 |
| SYK | 5 | 0.011905124 | 0.298165138 | 0.7 |
| PIK3CG | 5 | 0.001606511 | 0.298165138 | 0.8 |
| AHR | 4 | 0.164583596 | 0.391566265 | 0.5 |
| CALM1 | 4 | 0.076625548 | 0.323383085 | 0.166666667 |
| NOS3 | 4 | 0.043838349 | 0.333333333 | 0.333333333 |
| CDK4 | 4 | 0.031810897 | 0.320197044 | 0.333333333 |
| CHEK1 | 4 | 0 | 0.255905512 | 1 |
| CYP1B1 | 4 | 9.62E-04 | 0.256916996 | 0.666666667 |
| CYP17A1 | 4 | 0.002485557 | 0.283842795 | 0.833333333 |
| CYP19A1 | 4 | 0.150333605 | 0.355191257 | 0.5 |
| GSTA1 | 4 | 1.60E-04 | 0.256916996 | 0.833333333 |
| PDPK1 | 4 | 0 | 0.274261603 | 1 |

**Figure S4** The top targets in the PPI network as ranked using the cytoHubba plug in network analyzer. The higher degree value is represented by colors ranging from purple to blue.

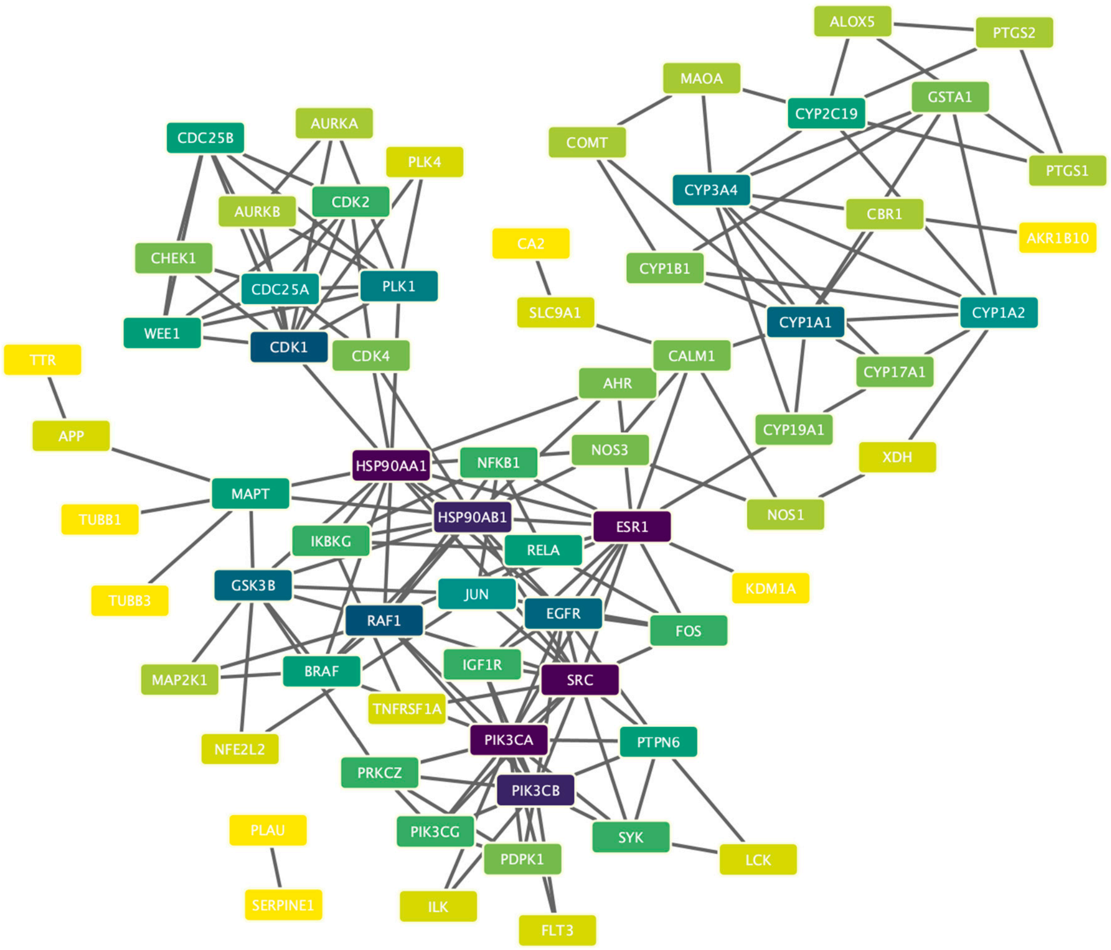
ALOX5
PTGS2
MAOA
GSTA1
CYP2C19
COMT
PTGS1
CDC25B
AURKA
PLK4
CYP3A4
CBR1
AKR1B10
AURKB
CDK2
CA2
CHEK1
CYP1B1
CDC25A
PLK1
SLC9A1
CYP1A1
CYP1A2
WEE1
CDK1
CDK4
CALM1
TTR
CYP17A1
AHR
APP
CYP19A1
XDH
NOS3
HSP90AA1
NFKB1
MAPT
NOS1
TUBB1
HSP90AB1
ESR1
IKBKG
RELA
KDM1A
GSK3B
TUBB3
JUN
EGFR
RAF1
FOS
IGF1R
MAP2K1
BRAF
SRC
TNFRSF1A
NFE2L2
PIK3CA
PTPN6
PRKCZ
PIK3CB
PLAU
PIK3CG
SYK
PDPK1
LCK
SERPINE1
ILK
FLT3
